# Single Cell Analysis Reveals the Stochastic Phase of Reprogramming to Pluripotency Is an Ordered Probabilistic Process

**DOI:** 10.1371/journal.pone.0095304

**Published:** 2014-04-17

**Authors:** Kyung-Min Chung, Frederick W. Kolling IV, Matthew D. Gajdosik, Steven Burger, Alexander C. Russell, Craig E. Nelson

**Affiliations:** 1 University of Connecticut Department of Molecular and Cell biology, Storrs, Connecticut, United States of America; 2 University of Connecticut Department of Computer Science and Engineering, Storrs, Connecticut, United States of America; Baylor College of Medicine, United States of America

## Abstract

Despite years of research, the reprogramming of human somatic cells to pluripotency remains a slow, inefficient process, and a detailed mechanistic understanding of reprogramming remains elusive. Current models suggest reprogramming to pluripotency occurs in two-phases: a prolonged stochastic phase followed by a rapid deterministic phase. In this paradigm, the early stochastic phase is marked by the random and gradual expression of pluripotency genes and is thought to be a major rate-limiting step in the successful generation of induced Pluripotent Stem Cells (iPSCs). Recent evidence suggests that the epigenetic landscape of the somatic cell is gradually reset during a period known as the stochastic phase, but it is known neither how this occurs nor what rate-limiting steps control progress through the stochastic phase. A precise understanding of gene expression dynamics in the stochastic phase is required in order to answer these questions. Moreover, a precise model of this complex process will enable the measurement and mechanistic dissection of treatments that enhance the rate or efficiency of reprogramming to pluripotency. Here we use single-cell transcript profiling, FACS and mathematical modeling to show that the stochastic phase is an ordered probabilistic process with independent gene-specific dynamics. We also show that partially reprogrammed cells infected with OSKM follow two trajectories: a productive trajectory toward increasingly ESC-like expression profiles or an alternative trajectory leading away from both the fibroblast and ESC state. These two pathways are distinguished by the coordinated expression of a small group of chromatin modifiers in the productive trajectory, supporting the notion that chromatin remodeling is essential for successful reprogramming. These are the first results to show that the stochastic phase of reprogramming in human fibroblasts is an ordered, probabilistic process with gene-specific dynamics and to provide a precise mathematical framework describing the dynamics of pluripotency gene expression during reprogramming by OSKM.

## Introduction

Methods of reprograming somatic cells to a pluripotent state (iPSC) have enabled the direct modeling of human disease and ultimately promise to revolutionize regenerative medicine [1], [2]. While iPSCs can be consistently generated through viral infection with the Yamanaka Factors OCT4, SOX2, KLF4, and c-MYC (OSKM) [3], infected cells rapidly become heterogeneous with significant differences in transcriptional and epigenetic profiles, as well as developmental potential [4]–[8]. This heterogeneity, the low efficiency of iPSC generation (0.1–0.01%) and the fact that many iPSC lines display karyotypic and phenotypic abnormalities [9]–[11] has hindered the production of iPSCs that can be used safely and reliably in a clinical setting. A thorough mechanistic understanding of the reprogramming process is critical to overcoming these barriers to the clinical use of iPSC.

In the past several years, ChIP-seq and RNA-Seq experiments have revealed ensemble gene expression and epigenetic changes that occur during reprogramming by OSKM, and have greatly enhanced our understanding of the process [2], [12]–[15]. These studies require the use of populations of cells comprised of heterogeneous mixtures undergoing reprogramming (0.01–0.1% of which will become iPSC) or stable, partially reprogrammed self-renewing lines arrested in a partially reprogrammed state, unlikely to ever become iPSCs without additional manipulation [5]–[8]. Because these techniques rely on either the ensemble properties of mixed populations, or upon the analysis of cell lines arrested at partially reprogrammed states that may not be representative of normal intermediate steps in a functional reprogramming process, they have limited ability to reveal the changes that appear to be essential to successful reprogramming.

Longitudinal single-cell imaging studies provide a powerful complement to ensemble, population level analyses. Live imaging studies have identified a number of key morphological and cell cycle related changes that occur during reprogramming to iPSC [16], [17]. These observations suggest that an ordered set of phenotypic changes precede acquisition of the fully pluripotent state [13]. However, these studies are necessarily limited in their molecular-genetic resolution, and they provide little insight to the transcriptional changes accompanying key morphological and developmental transitions in the reprogramming process.

Recently, a single-cell transcriptional analysis of reprogramming of mouse fibroblasts by OSKM revealed that reprogramming proceeds in two major phases: an early stochastic phase followed by a rapid “hierarchical” phase [18]. While the latter phase appears deterministic and is characterized by the coordinated expression of pluripotency genes in an ordered fashion, the early phase exhibits apparently random gene expression patterns that persist through the majority of the process [18], [19]. This conclusion is further supported by two key pieces of evidence from other studies: 1) transgenic OSKM activity is required for the majority of the reprogramming process, indicating that most of this process is not governed by the concerted action of the endogenous pluripotency gene regulatory network (GRN) [16], [20], [21]; and 2) a mechanistically undescribed period of variable ‘latency’ of cells in the stochastic phase results in significant temporal variability in the appearance of fully reprogrammed iPSC colonies [22]. Some insight to pluripotency gene activation during the stochastic phase was provided by a recent study in mouse fibroblasts that describes the ‘gradual activation of pluripotency genes’ between the initial response to OSKM induction and the activation and stabilization of the pluripotency GRN [23]. Together, these findings suggest that the stochastic phase is a major rate-limiting step in the reprogramming process, but provide little mechanistic insight into the molecular underpinnings of these events. In addition, it has not yet been determined how these findings translate to the reprogramming of human cells, which will be required prior to clinical application of iPSCs.

Several studies have attributed the protracted stochastic phase to the requirement for extensive chromatin remodeling during reprogramming [24], [25]. These changes involve the complex coordination of factors to deposit and remove histone modifications and DNA methylation at specific loci to achieve a pluripotent epigenetic state. The need to reset the epigenetic landscape appears to delay the coordinated activation of the pluripotency GRN and is likely to be a major barrier to rapid and efficient reprogramming. Indeed, it has been shown that OSKM binding in the early stages of reprogramming is greatly impeded by the presence of repressive chromatin, and initial binding is largely restricted to existing open chromatin domains [2], [14], [15], [26], [27]. Subsequent remodeling of somatic cell chromatin clearly occurs, but the order and mechanism of remodeling events during the stochastic phase is not fully understood. Accurate mapping of gene expression dynamics during the stochastic phase can provide a framework for the molecular dissection of these rate-limiting events in reprogramming.

In this study we perform single-cell transcript analysis of MRC-5 human lung fibroblasts undergoing reprogramming by OSKM and find that cells appear to follow two trajectories: one toward an ESC-like state (the “productive” trajectory) and the other away from both ESC and fibroblasts (the “alternative” trajectory). These trajectories can be differentiated by the concerted consolidation of expression of a suite of chromatin modifiers in cells entering the productive trajectory and the down-regulation of these same genes in cells entering the alternative trajectory. By analyzing the dynamics of gene expression changes along the productive trajectory (toward pluripotency) we demonstrate that changes in gene expression in the stochastic phase of reprogramming are not simply gradual and random; rather, genes are activated and inactivated at specific points during the progression from fibroblast to iPSC. Coupling single-cell transcript profiling with mathematical modeling we show that the gradual acquisition of pluripotency gene expression during reprogramming occurs as an ordered, probabilistic, gene-specific process that shows no signatures of interdependence between genes. This finding is consistent with the hypothesis that gene-specific chromatin states in the starting cells control gene activation dynamics during the reprogramming process. Our map of reprogramming also provides a robust model that can be used to dissect the precise mechanisms and chromatin modifications that limit the rate and efficiency of conversion of somatic cells to iPSC. This work represents a rigorous single cell transcript analysis of the reprogramming process in human cells and lays the foundation for the precise measurement and mechanistic dissection of this critical rate-limiting step in reprogramming.

## Results

### Experimental Design

**Figure 1:**
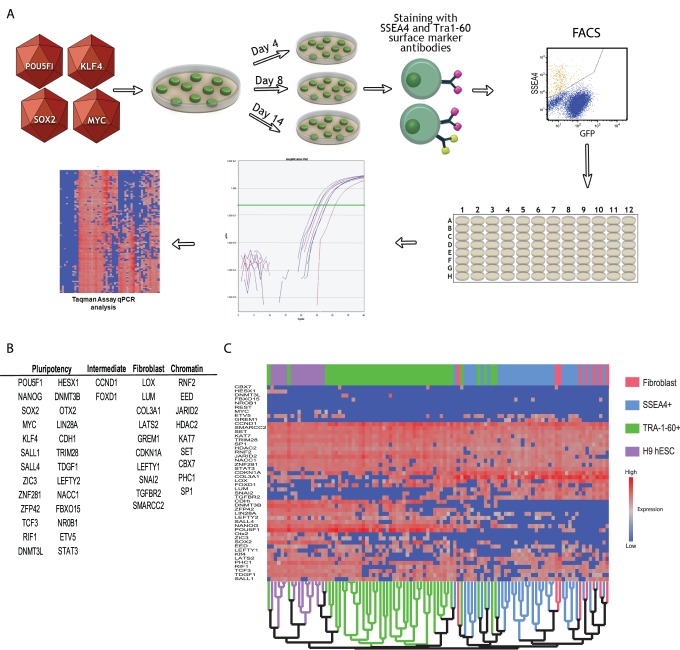
Schematic representation of the pipeline used to isolate and analyze single cells undergoing OSKM-mediated reprogramming. A) Cells were infected with OSKM (MOI = 5) and cultured for 4, 8 or 14 days prior to harvest. Cells were then singularized and stained with SSEA4 and TRA-1-60 antibodies and subjected to FACS. SSEA4^+^/TRA-1-60^−^ (SSEA) and SSEA4^+^/TRA-1-60^+^ (TRA-1-60) single cells were sorted directly into lysis buffer in 96-well plates followed by RT and linear pre-amplification. Amplified cDNA samples were used for Taqman qPCR analysis of 48 genes on an Applied Biosystems 7900 HT real time machine and data analysis was performed in JMP. B) Table of the 48 gene panel used for qPCR analysis, categorized as fibroblast-associated, pluripotency-associated, intermediate marker or chromatin modifier gene. C) Unsupervised hierarchical clustering analysis illustrating the effective isolation of single cells by FACS for SSEA4 and TRA-1-60 surface markers. While some overlap is observed between the two populations, they are largely transcriptionally separable. GFP^+^-only and CDH1^+^ populations have been excluded for illustrative purposes.

In this report we combine qualitatively and quantitatively robust single-cell transcript profiling [28] with FACS to measure the progression of individual MRC-5 human fetal lung fibroblasts through the reprogramming process. To make our results as broadly relevant as possible we used viral delivery of the OSKM transgene cocktail, the most widespread method applied to human cell reprogramming [29], [30]. At select time points after transduction, cells were dissociated, stained, analyzed and collected by FACS. FACS markers used in this study include GFP (virus derived), αSSEA4, αTRA-1-60, and αCDH1 (see Materials and Methods). These markers were essential and allowed for enrichment of the rare cells exhibiting hallmarks of productive reprogramming. For example, SSEA4 and TRA-1-60 routinely provide ∼30 and 3,000 fold enrichment, respectively (data not shown). While very few SSEA4+ cells are likely to become true iPSCs, they provide a measurement of cells that have begun to exit the fibroblast in response to OSKM transduction. In contrast, isolation of TRA-1-60+ cells later in reprogramming (Day 14) is likely to yield a large number of cells destined to become iPSC. In fact, >90% of these cells remain TRA-1-60+ after sorting and subsequent culture and this stability of the TRA-1-60+ phenotype has been shown to be a major determinant for the potential of cells to become iPSC [31]. Single cells with defined FACS phenotypes were collected into cell lysis buffer and subject to single-cell RT-qPCR as previously described [28] (Figure 1A and Figure S1). Throughout the course of this study we isolated and pre-screened 576 cells in total, using 172 cells that passed quality control for our final analysis (see Materials and Methods and Table S3). This includes many partially reprogrammed cells, as well as an un-transduced set of MRC-5 fibroblasts and H9 human embryonic stem cells (H9-hESC), which represent the beginning and end states of the process, respectively (for full dataset see Table S4).

In order to monitor progress toward pluripotency, and away from the fibroblast state, we assembled a 48-gene qPCR (Table S1) panel including genes expressed in fibroblasts [17], , a large number of genes involved in the maintenance of pluripotency (including various chromatin modifiers) [12], [34]–[36] and genes previously suggested to be intermediate markers of the reprogramming process [37], [38]. For a complete list of qPCR markers see (Figure 1B and Figure S2). Initial visualization of the full dataset by unsupervised hierarchical clustering reveals that our FACS sorting strategy, and qPCR marker panel, isolates statistically separable populations that capture a range of transcriptional phenotypes between the fibroblast and pluripotent states (Figure 1C). We then performed a series of statistical analyses to: 1) describe probable trajectories followed by OSKM-infected cells; 2) measure the progress of cellular transcriptional profiles toward a pluripotent transcriptional phenotype; and 3) determine the order of gene activation during the reprogramming process.

### Mapping the Trajectory of OSKM-Infected Cells Throughout Reprogramming

**Figure 2:**
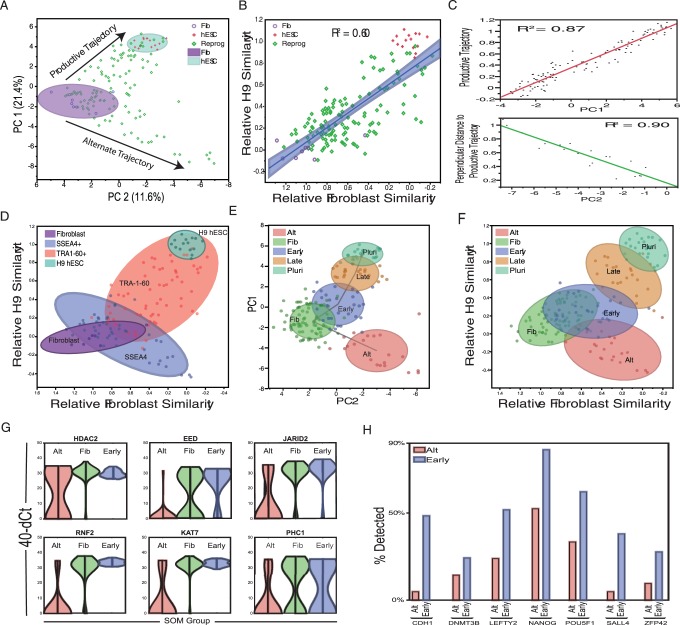
Mapping the trajectories of OSKM- infected cells. A) Principle Components Analysis (PCA) shows the two trajectories followed by OSKM-infected cells. One productive trajectory leading away from the starting fibroblast population (purple oval) and towards the hESC group (teal oval) and a second, orthogonal trajectory leading away from both fibroblast and hESC, denoted as the “alternate trajectory”. B) Regression analysis showing the linear nature of the productive trajectory. C) Correlation analysis between PC1 and the productive trajectory (C, top panel) and PC2 and the perpendicular distance to the productive trajectory. D) Mapping of cell types onto a Euclidean distance graph shows the broad range of transcriptional phenotypes observed for SSEA4+ (blue oval) and TRA-1-60+ (pink oval) FACS-sorted cells. Also included are untransfected MRC-5 fibroblasts (purple oval) and pluripotent H9 hESC cells (teal oval). Self-Organizing Map (SOM) analysis identifies transcriptionally separable groups within our dataset in PCA (E) and Euclidean (F) space. This includes 4 groups along the productive trajectory (Fib, Early, Late and Pluri) as well as one group comprised of cells in the alternate trajectory (Alt). G) Violin plots comparing expression of chromatin modifier genes between the Alt (red), Fib (green) and Early (blue) groups. Gene expression levels are plotted on the y axis, with the width of the graph representing the prevalence of cells at a given expression level. H) Bar graph illustration differences in pluripotency gene expression between the Alt and Early groups.

As a first step in visualizing our single cell transcription dataset, we used principal components analysis (PCA) to assess the complexity and major sources of variation in gene expression between all cells collected in our study. This analysis reveals that the first two PCA dimensions account for 33.1% of the observed variation, where PC1 primarily represents a cell’s distance from hESC, and PC2 primarily captures distance from fibroblasts (Figure 2A). In addition, these two axes appear to represent distinct trajectories followed by cells transduced with OSKM The first is a roughly linear productive trajectory between the fibroblast and hESC groups (R^2^ = 0.60, Figure 2B) and the second is an orthogonal trajectory leading away from fibroblast but not towards a pluripotent phenotype (herein referred to as the alternate trajectory, or ALT). Because the productive and alternate trajectory are well correlated with the PC1 and PC2 dimensions respectively (Figure 2C) and capture much of the variation in our dataset, we developed a metric to analyze our data in a 2-dimensional Euclidean space that maps each cell’s distance (relative similarity) to the centroids of both the Fibroblast and hESC groups. In addition, we construct a Euclidean diagonal between Fibroblast and hESC which we term the “reprogramming progression axis”. This axis serves as a useful measurement of a given cell’s progression towards pluripotency and is a metric used in all subsequent analysis presented here.

It is important to note that our analysis constructs likely reprogramming trajectories by sampling partially reprogrammed cells. This approach is common among many efforts to sample dynamic processes and is particularly ubiquitous in attempts to dissect the reprogramming process [19], [24], [39]. We apply the standard parsimonious assumption that the shortest path defined by these samples represents the most likely trajectories of the process. One caveat of this approach is that we cannot exclude the possibility that progression within the observed state-space is non-linear, and may be complex and/or cyclical. These possibilities will need to be ruled out with longitudinal live cell studies beyond the scope of this work. Another important consequence is that while cells clearly take time to traverse the trajectory, we do not expect progress along a trajectory to have a linear relationship with time. However, progress may be loosely thought of as a surrogate for time but should not be strictly interpreted as such.

Interestingly, when mapping the FACS-sorted phenotypes onto our Euclidean similarity graph we noticed that, while SSEA4 and TRA-1-60 appear in the expected order (SSEA4^+^ before TRA-1-60^+^), the SSEA4^+^ and SSEA4^+^/TRA-1-60^+^ populations exhibit considerable transcriptional heterogeneity (Figure 2D). SSEA4 positive cells are found in both the productive and alternative trajectories suggesting that, while SSEA4 may be a reliable marker of exit from the fibroblast state, it does not necessarily indicate that cells have moved toward a pluripotent transcriptional phenotype. Even more pronounced is the diversity of TRA-1-60 positive cells. The transcriptional phenotype of these cells extends from a nearly fibroblast-like profile, to a nearly ESC-like profile. The extremely high degree of transcriptional heterogeneity we observe, even within well-defined and widely utilized FACS profiles, underscores the utility of single cell analysis to dissect fine differences in gene expression between partially reprogrammed cells.

With the phenotypic diversity of commonly utilized cell surface markers in mind, we utilized a Self-Organizing Map (SOM) to identify separable groups along the two previously described reprogramming trajectories in both PCA and Euclidean space (Figure 2E and F, respectively). Four of these groups (Fib, Early, Late and Pluri) lie along the productive trajectory from Fibroblast to ESC and the fifth encompasses cells in the alternate trajectory. It is important to note that while these groups can be statistically distinguished from one another, we do not believe these represent discrete stages in the reprogramming process. Further inspection reveals that progression along the productive trajectory is characterized by the consolidation of chromatin modifier expression, an increased probability of pluripotency gene expression, a progressive decrease in the expression of fibroblast markers and transient expression or repression of predicted intermediate markers [22], [38]. Among the earliest distinctions between the productive and alternate trajectories (Early vs Alt) is the induction of chromatin-modifying enzyme expression. While many of these genes are expressed at low levels in fibroblasts, they are coordinately up-regulated in the “Early” group, and become expressed at uniformly high levels in all cells progressing towards pluripotency. In contrast, cells in the alternate trajectory down-regulate or eliminate expression of these genes (Fig. 2G). In addition, “Alt” cells fail to upregulate the expression of early pluripotency genes (Figure 2H) and are found at all of the time points examined, suggesting that these cells are unlikely to be on a trajectory that ultimately leads to pluripotency. Because “Alt” cells appear to be following an orthogonal trajectory that may lead to fates unrelated to ESC (such as transformation or apoptosis [39], [40]) they were excluded from further analysis of the productive reprogramming trajectory.

Taken together these data indicate that OSKM infected cells exit the fibroblast state along two distinct trajectories, and that the upregulation of chromatin modifiers marks a key early step towards successful reprogramming. The rapid upregulation of chromatin modification genes is consistent with the need for extensive chromatin remodeling prior to establishment of the endogenous pluripotent GRN [2], [41], [42].

### Mapping Coarse Changes in Gene Expression along the Productive Trajectory

**Figure 3:**
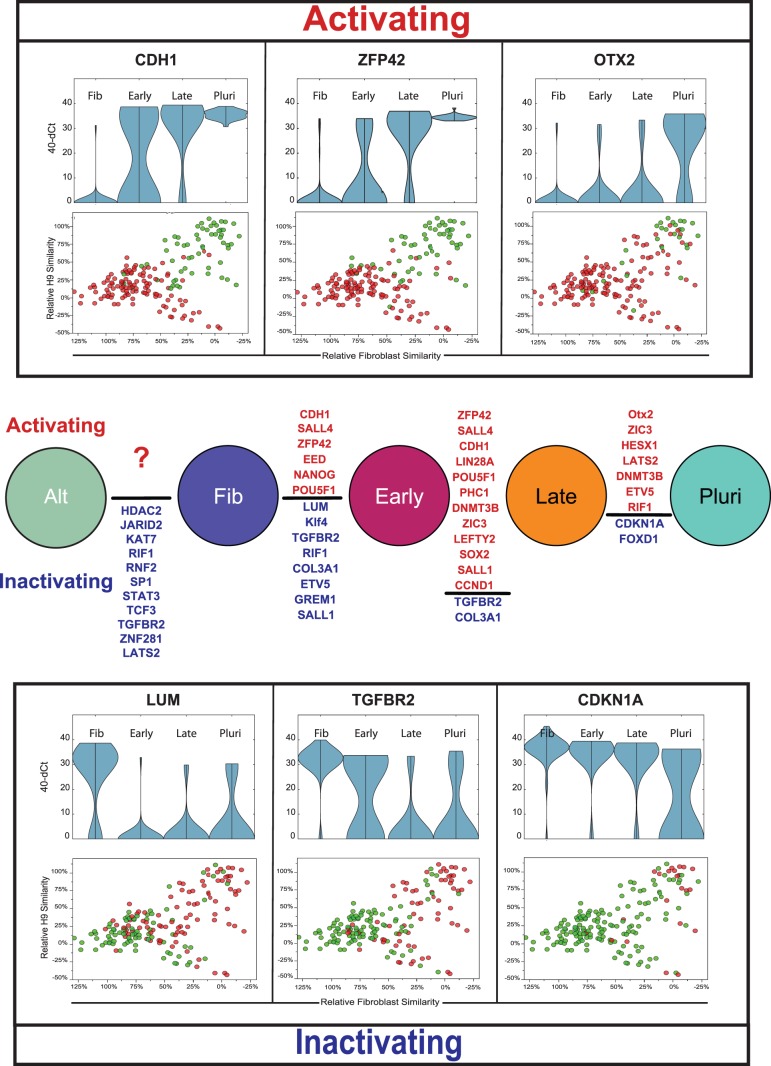
(Middle panel) Tukey-Kramer test results showing significant increases or decreases in gene expression between the groups identified in the PC-SOM analysis (p>0.05). Genes are ranked in order of significance from highest to lowest. Violin and bubble plots (above and below) show qualitative and quantitative changes (respectively) in per-cell gene expression for the genes with the greatest change between groups. Top panel shows genes whose level and probability of expression undergo an “activating” effect during reprogramming, while genes with decreased probability of expression during reprogramming are labeled “inactivating” and shown in the bottom panel.

In order to provide a rough benchmark for other literature examining transcriptional changes in ensemble samples of partially reprogramed cells, we identified quantitative expression differences between SOM groups along the productive trajectory (Figure 3). It is clear from this data that specific changes in gene expression occur along different portions of the trajectory, which suggests an underlying order to the gradual acquisition of pluripotency gene expression during the reprogramming process. However, closer analysis reveals that there does not appear to be tight covariance between genes activated along the progression toward pluripotency. Representative bubble plots illustrating transcript presence and absence (Figure 3 and Figure S2) show that genes being activated during reprogramming exhibit a period of heterogeneity in transcript detection prior to being detected in all cells approaching pluripotency. Quantitative analysis of gene expression levels also supports this finding (Figure 3, Figure S3). These plots depict gene expression levels on the y-axis, overlain with a distribution graph showing the range of expression values within the population. A unimodal distribution indicates uniform expression around a mean within the population, whereas a bimodal distribution demonstrates a transcriptionally heterogeneous population (e.g. high/low) for the gene in question. Nearly all the genes in our study exhibit this bimodal behavior at some point along the reprogramming trajectory, before achieving a unimodal distribution as they approach the fully reprogrammed state, however the point of bimodality varies in a gene-specific manner. These findings demonstrate that the activation or inactivation of gene expression during reprogramming proceeds through a probabilistic intermediate step, resulting in transcriptionally heterogeneous cell populations, and that the timing of this transition occurs with gene specific dynamics.

In order to scan for potential differences in reprogramming gene expression dynamics between species (mouse and human) we processed our data so that it would be roughly comparable to that generated by Polo et al [23]. As in the present study, Polo and coworkers used FACS to isolate and measure the transcriptional profiles of a large number of partially reprogrammed mouse fibroblasts and clustered genes based on their expression dynamics. We compared these clusters to the dynamics of the human orthologs [12], [34] represented in our dataset (Figure S4). While high-resolution comparison was not possible with the publically available mouse data, most genes shared between datasets appear to exhibit similar dynamics in the stochastic phase. That is, early mouse genes change expression early in the human trajectory, while late genes change later in the trajectory. However, despite the coarse limits of resolution in this comparison, several genes, including NANOG, LIN28A, POU5F1 and STAT3, appear to change at different stages of the reprogramming process in these two species. These disparities, while requiring more direct comparison and detailed confirmation, are consistent with distinct differences between regulation of the pluripotent state in mouse and human cells as well as probable differences in the starting chromatin state of loci in mouse and human fibroblasts.

### Reprogramming is a Loosely Ordered Probabilistic Process Effectively Modeled by Gaussian Distributions

Our observation that distinct transcriptional differences exist between PC-SOM clusters indicates that gene expression changes during the stochastic phase of reprogramming appears to occur in an ordered fashion. However, the coarse grained nature of this differential analysis between statistically identifiable, but not necessarily biologically relevant groups, provides little insight to the exact nature of the order of gene expression dynamics during the stochastic phase. In particular, we wanted to address two specific questions: 1) Is the acquisition of pluripotency gene expression random and gradual, with all genes approaching a pluripotent profile at a uniform rate over the course of the process?; and 2) Is there sub-structure within the patterns of gene activation that would suggest the activation of modules within the pluripotency GRN? We addressed these questions by differentiating between null and alternative hypotheses (in the form of distribution models) predicting gene expression frequencies along the reprogramming trajectory from MRC-5 to H9-ESC and comparing these to what we observe in our experiments.

**Figure 4:**
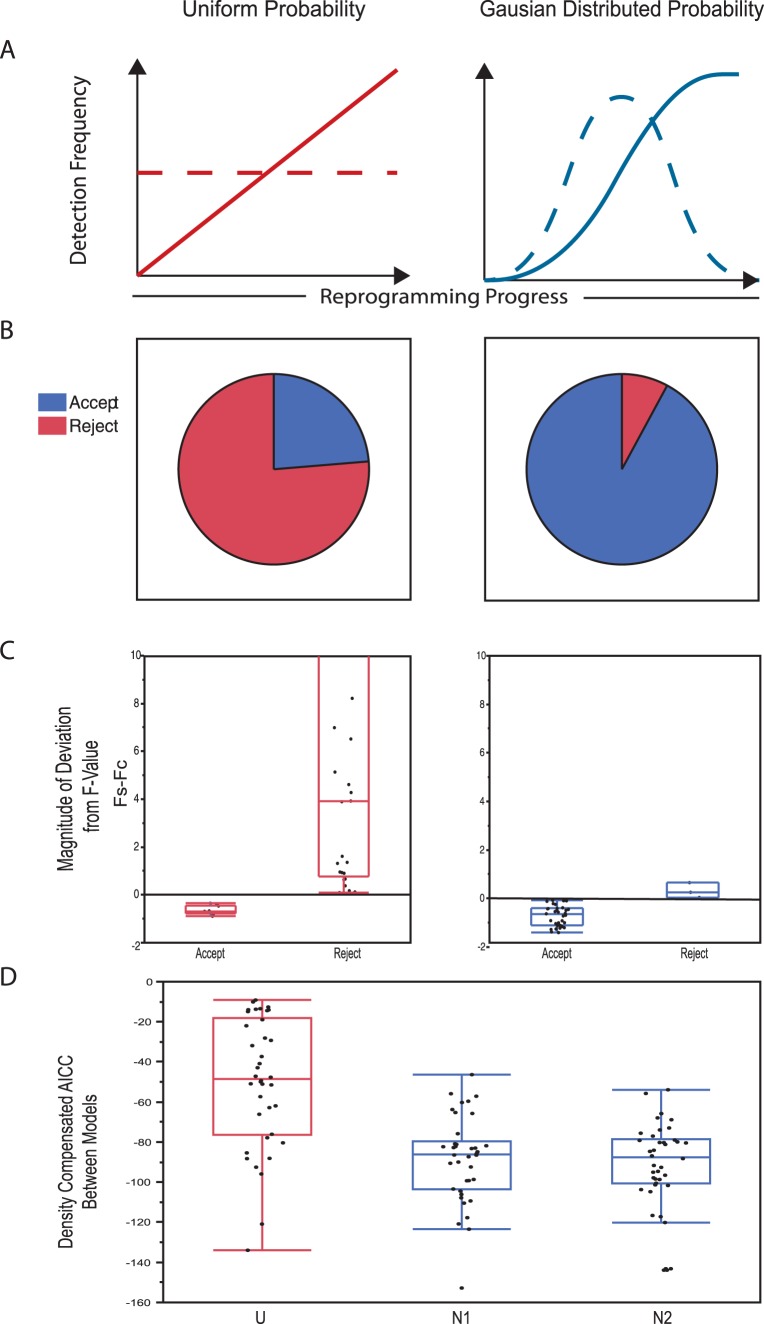
Rejection of a uniform model and justification of modeling using Gaussian distributions. (A) Predicted outcomes of gene expression probabilities associated with uniform (left panel) or Gaussian (right panel). Uniform and Gaussian probability distributions (dashed line) give rise to cumulative probabilities (solid line) that describe the population of cells at a given point in time. A Uniform probability results in the gradual activation/inactivation of a gene throughout the process, while Gaussian distributions suggest a bias in expression change towards a particular point in the process. (B) Pie charts showing the relative number of genes that accept or reject the Uniform (left panel) or Gaussian model (right panel) as determined using an F-statistic test. The strength with which these genes accept or reject each model is shown in (C). (D) Comparison of AICC value for all genes between the Uniform model and a Gaussian model using one or two normal distributions. While considerable improvement is observed for the Gaussian vs Uniform model, the addition of a second normal distribution does not dramatically improve model fit.

In order to formally address the first question we modeled random gradual change in gene expression by assigning each fibroblast and pluripotency marker a uniform rate (probability) of change along the trajectory from MRC-5 to H9-ESC that would result in predicted gene expression frequencies that match the observed frequencies at the start (MRC-5) and end (H9-ESC) of the process [23]. In contrast, our alternative hypothesis was that genes change expression at specific stages of the process; in other words, gene expression during the stochastic phase is *ordered*. This alternative scenario was modeled by fitting Gaussian probability distributions to each gene such that the probability distribution was centered at the point of greatest change in gene expression frequency along the reprogramming trajectory. In order to model the behavior of transient genes, and to help calibrate differences between goodness of fit between models, we also built more complex models with two probability distributions, which allowed us to model genes that change expression at two points in the process. Changes in gene expression frequency predicted by our null model are linear, while the alternative model with one probability distribution predicts sigmoidal changes and the two distribution model allows for more complex dynamics of change in gene expression frequency, such as transient activation or inactivation. The goodness of fit of each model to our observed data was then measured for each gene in both PCA and Euclidean space using an F-test statistic. Because goodness of fit typically scales with the number of parameters in a model, the Gaussian models were penalized for added parameters using a corrected Akaike Information Criterion (AIC, see Materials and Methods). The results of these tests can be found in (Figure 4A–D and Table S2).

As demonstrated in Figure 4B, the vast majority of genes reject the null hypothesis (F-statistic>F-Critical) in favor of a Gaussian model. Note that many genes that reject the null hypothesis do so very strongly, while the few genes that better fit linear dynamics do so only marginally (Figure 4C). In addition, most genes that do not reject the uniform model exhibit little or no change over the course of reprogramming or have noisy expression profiles. Both of these observations suggest that most gene expression changes occurring during the stochastic phase are not simply gradual acquisition of an ESC-like expression frequency, rather they turn on and off at specific points in the process.

To further assess the confidence with which random change (uniform probability distribution) in gene expression during the stochastic phase can be rejected by our models (Gaussian probability distribution) is to compare the explanatory power of each model, as adjusted for the additional parameters required in each more progressively complex scenario. Figure 4D shows that while one normal distribution significantly improves AIC (lower is better), two normal (or even three normal - data not shown) do not add much explanatory power. One exception is for genes that exhibit transient expression changes, the fits for which are shown in Figure S6. For this reason, we suggest that gene expression dynamics during the stochastic phase are best described as events occurring at specific points in the process, where most gene’s expression dynamics are well described by a single normal probability distribution centered at the point of maximal rate of change. Genes that change at very specific points in the process have very tight probability distributions, while genes with less precise dynamics display broader probability distributions (approaching the uniform distribution of our null model).

**Figure 5:**
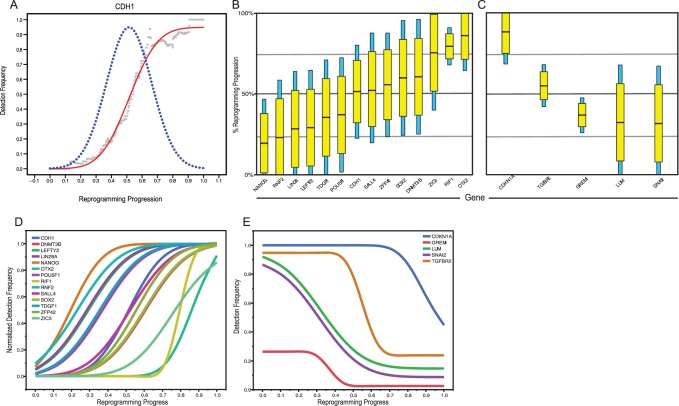
(A) Goodness of fit of a Gaussian model using activation of the CDH1 gene as an example. Gaussian distributions are represented as box and whisker plots for activating (B) and inactivating (C) genes. Yellow boxes and blue whiskers represent the 50% and 95% confidence intervals of the normal curve respectively, with the means shown as black lines. Cumulative distributions derived from the Gaussian model are overlaid for genes that are activated (D) or inactivated (E) during the course of reprogramming.

In order to compare dynamics between genes, we modeled each gene in our study using single Gaussian probability distributions as described above. All model fits are illustrated in the Figures S5. One example fit is illustrated for CDH1 in Figure 5A. In this figure the black dots represent measured expression frequencies of CDH1 in sliding windows along the inferred reprograming trajectory. The red curve shows gene expression dynamics modeled as a Gaussian probability distribution fit to the experimental data and the blue line illustrates expression frequencies predicted by that probability curve.

When the dynamics of several genes are compared in one graph (Figure 5B–E) it is readily apparent that: 1) *genes are activated or inactivated at different points during the reprogramming process*; 2) *genes have specific stringencies in their activation dynamics (some genes change at fairly specific stages, while others change over almost the entire course of the process)*; and 3) *there is considerable overlap in the expression probabilities of individual genes*. Most genes are activated or repressed with diffuse dynamics, while several (NANOG, CDH1, ZFP42, ZIC3 and OTX2) change at more specific stages of the reprogramming process. The diffuse dynamics and broad windows of activation observed for most pluripotency markers is consistent with the longitudinal observation that the expression of the surface antigens SSEA4 and TRA-1-60 in iPSC colonies are not strongly predictive of successful reprogramming events [20], [37]. Taken together, this data strongly supports the hypothesis that rather than being a strictly ordered or strictly random process, the stochastic phase of reprogramming is an ordered probabilistic process. *Seen in this light, prior ordered and random models can be coherently united* [43] [44] [19].

### Changes in Pluripotency Gene Expression during the Stochastic Phase do not show Hallmarks of Activation of the Pluripotency Gene Regulatory Network

**Figure 6:**
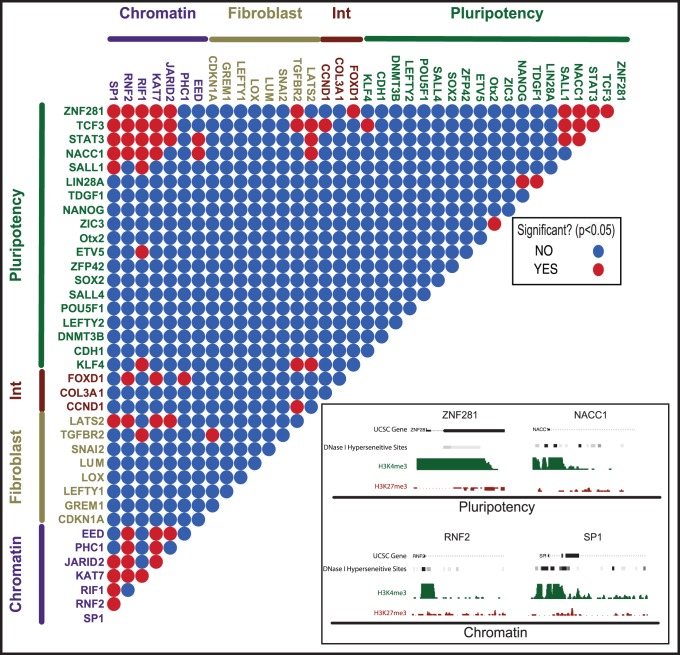
Cells undergoing reprogramming do not show hallmarks of activation of the pluripotency GRN. Heat map shows background-corrected Pearson’s correlation coefficients for all genes in our dataset, excluding NR0B1 and REST (due low detection frequency). Significant correlations (red dots) are primarily observed for chromatin genes, while the majority of pluripotency genes show no significant correlations (blue dotes). A small group of pluripotency genes with significant correlations exhibit an open chromatin state in the starting cell type indicated by H3K4me3 promoter methylation and DNase hypersensitivity (Inset).

Having observed ordered dynamics in the stochastic phase, we sought to determine if there was any indication that this order might arise from the partial activation of the endogenous pluripotency GRN. Current models suggest that partially reprogrammed cells enter a late, rapid deterministic phase that is controlled by activation of the endogenous pluripotency GRN and may be marked (in mouse cells) by the activation of the endogenous Sox2 locus [20], [46]. Alternatively, order could emerge gradually or piecemeal during the stochastic phase. A hallmark of concerted gene regulation as exerted by a GRN, is strong correlation (or anti-correlation) between gene expression patterns [18], [19], [23]. Our model provides a powerful way to detect correlated gene expression that lies above the background correlations inherent during reprogramming (i.e. pluripotency markers all become expressed in fully reprogrammed cells). In this case, our null hypothesis is that during the stochastic phase there is no dependency between genes and that all correlation between gene expression in individual cells results simply from the increase in frequency of pluripotency markers as cells approach an ESC-like transcriptional profile. Our alternative hypothesis is that some pluripotency genes may be co-regulated (or cross-regulate) during the stochastic phase and would thus display higher than background levels of co-expression (as measured by correlation). To test these hypotheses we used the probability profiles of each gene to generate a simulated data set in which gene expression is determined only by the probability profile of each gene, with no dependencies between genes. The resulting dataset accurately recapitulates the individual dynamics of each gene in our dataset, and provides pairwise correlation values that are solely dependent upon the convergence of all pluripotency markers on uniform expression in ESC. We then compared pairwise correlations between genes in this background data set with the real correlations observed in our single-cell transcript data (Figure 6).

Interestingly, the only correlations we find rise above background expectations occur between a set of chromatin regulators that distinguish between entry into the productive trajectory and entry into the alternative trajectory (Figure 6). This coordinated activity is likely the result of activation of the c-MYC GRN, which is known to be activated upon OSKM induction, and is largely limited to genes with a permissive chromatin state in fibroblasts as is the case for many chromatin modifier genes [45], [46] (Figure 6, inset). In contrast, none of the correlations between members of the pluripotency GRN rise above background expectations, despite their overall increase in expression frequency as cells approach an ESC-like expression profile. We therefore accept the null hypothesis: that despite the ordered activation of genes in the pluripotency GRN during the reprogramming process, there is no evidence for gradual or modular activation of the pluripotency GRN during the stochastic phase of reprogramming. An important corollary that follows from this result is that *the dynamics of gene activation during the stochastic phase appear to depend only upon the local properties of each gene*, rather than the sequential activation of precursors in the GRN. Of course, the numbers of genes we analyze in our study somewhat limits the power of this analysis, and a more comprehensive single-cell study measuring many more genes might uncover obligate relationships between genes that are not apparent in our core pluripotency GRN gene set.

## Discussion

**Figure 7:**
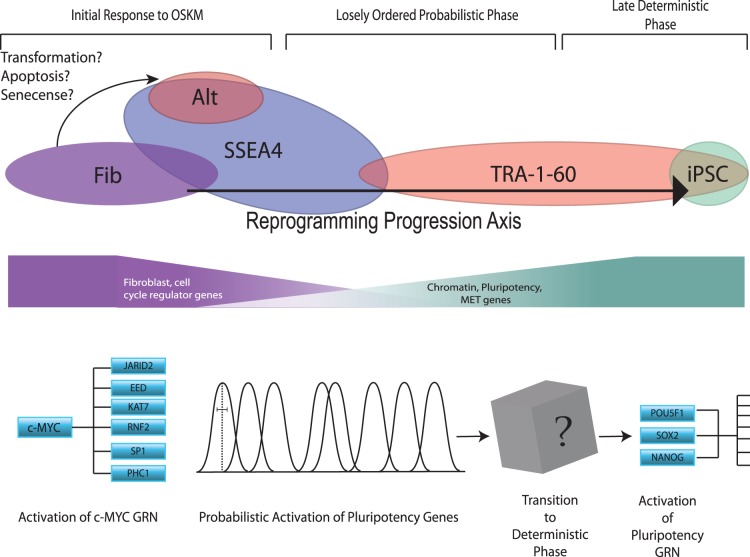
Combined models describing the trajectories and transcriptional phenotypes observed during somatic cell reprogramming. (Top panel) Two trajectories are observed for cells undergoing reprogramming by OSKM, a productive trajectory leading towards pluripotency and an alternate trajectory away from fibroblast but not towards a hESC phenotype. The productive trajectory is characterized by the expression of the surface markers SSEA4 early and TRA-1-60 late in the process, and in general, involves the down-regulation of fibroblast and cell cycle-associated genes and simultaneous up-regulation of chromatin modifier and pluripotency genes. Putting our results in the context of the current literature, we observe an early wave of gene induction involving chromatin modifying enzymes and other loci with an open chromatin state that is likely the result of cMYC and KLF4 activity at these promoters. This initial wave is followed by a period of independent probabilistic gene expression, which we have model using a series independent Gaussian distributions. This probabilistic phase of pluripotency gene activation will eventually lead to an as yet unknown event that allows transition into the deterministic phase and the subsequent acquisition of pluripotency.

In this study we present a rigorous single cell analysis of reprogramming in human cells and show that the stochastic phase of reprogramming of human fibroblasts by OSKM is an ordered probabilistic process which can be simply modeled using independent Gaussian distributions. An advantage of our approach lies in the fact that it makes no *a priori* assumptions about the progression of cells toward pluripotency, based on time or surface marker expression, both of which are poor indicators of reprogramming progress. In addition, the simplicity of our model and its exceptional fit to our observed expression dynamics provide a tractable framework for further dissecting the rate-limiting aspects of reprogramming. The results of this work also unify existing ordered and random models of the stochastic phase of reprogramming [16]–[18], [21], [22], [37] and are consistent with observations from both population level and single cell studies of gene expression changes during reprogramming [37], [38], [43]. The ordered nature of the stochastic phase is readily apparent in the distinct, gene-specific expression dynamics we observe during reprogramming, while the probabilistic nature of the process is evident in broad gene-specific expression dynamics over large portions of the reprogramming trajectory (Figure 5 and Figure 7), and the apparently independent control of gene expression dynamics during the stochastic phase (Figure 6). These findings are consistent with a recent study by Tanabe et al. [31] that suggests the TRA-1-60+ phenotype is unstable and transcriptionally heterogeneous and that stabilization of the TRA-1-60+ population is a critical rate limiting step in reprogramming. Note we suggest retaining the term “stochastic” for this phase of the reprogramming process, in that stochastic can be used to describe ordered probabilistic events, and does not necessarily imply complete randomness. The use of the term stochastic is especially appropriate given the independence of activation dynamics of key genes in the core pluripotency GRN.

One consequence of the independent activation of genes during reprogramming is that an extremely wide variety of cell states are present during the reprogramming process, which gives the overt appearance of disorder. Thus, while any given partially reprogrammed cell’s gene expression pattern may appear to be random, the probabilities of expression of individual genes are clearly biased towards specific points along the reprogramming trajectory. One implication of these findings is that any single marker is unlikely to be effective at determining the extent to which a given cell has been reprogrammed [37], [47].

We note that variations in the cell cycle could contribute to the transcriptional heterogeneity of a subset of genes in our dataset. However recent studies in hESC have shown that the transcription of genes associated with pluripotency does not fluctuate during the cell cycle [48], suggesting that cell cycle status is unlikely to have a major impact on our analysis of the activation of the pluripotency GRN. In addition, the persistence of cyclin transcripts throughout the cell cycle and their considerable post-transcriptional regulation in ESC’s [49], precludes strong inference of cell cycle status from transcriptional measurement of a single cell-cycle regulator.

Another possible source of transcriptional heterogeneity between partially reprogrammed cells in our cultures could be the delivery of O, S, K, and M on individual vectors (as is standard in widely utilized human reprogramming protocols). However the broad agreement of expression dynamics over the course of reprogramming between our results using individual viral delivery, and those reported by Polo et al using an inducible, polycistronic construct in a clonal cell line, suggests that viral heterogeneity does not fundamentally affect the order of gene expression dynamics, or the shape of the trajectory of cells undergoing the reprogramming process. Furthermore, the initial description of the highly heterogeneous nature of the stochastic phase by Buganim et al was also derived from data using clonal cells expressing OSKM from an inducible polycistronic OSKM construct. Thus, the stochastic nature of this phase does not appear to be a direct consequence of OSKM heterogeneity. However, these results do not rule out the possibility that each of the OSKM factors have distinct roles in various stages of the reprogramming process, nor that heterogeneity in OSKM content will be observed across the partially reprogrammed population of cells. Indeed, understanding the role of each factor in the reprogramming process and the critical window for the action of each represents an important goal of future work.

A likely explanation for the apparent lack of deterministic behavior during the stochastic phase may be the existence of as yet unidentified, gene-specific factors that restrict the rate of transcription activation by OSKM. One compelling candidate for these factors is the local chromatin architecture of the pluripotency genes in the starting somatic cell type. Indeed, epigenetic remodeling was implicated as a major rate limiting step in even the earliest days of somatic cell reprogramming using nuclear transfer [24], [25] and is almost certainly one of the most important probabilistic events limiting the rate and efficiency of reprogramming. Many reports have experimentally validated this hypothesis by demonstrating that global chromatin reorganization is critical for successful reprogramming [2], [14], [15], [27]. Because many of the required changes in chromatin state appear to occur in a slow and probabilistic fashion [50]–[52] it is likely that these changes limit the rate at which exogenous OSKM can activate the endogenous pluripotency GRN thus limiting the efficiency and speed of reprogramming and endowing the majority of the process with stochastic dynamics.

Our finding, that enhanced expression of chromatin modifiers is a hallmark of entry into productive reprogramming complements several studies demonstrating that successful reprogramming requires the gradual erosion of epigenetic barriers to activation of the pluripotency GRN by OSKM [2], [26], [27], [40], [53]. This event is likely governed by the activity of c-MYC, which together with KLF4, acts early in reprogramming to activate loci with permissive chromatin states, including many chromatin modifier loci in fibroblasts [14], [26]. In addition, many treatments known to enable chromatin remodeling have been shown to enhance the rate and/or efficiency of the reprogramming process [53]–[56], while, conversely, knocking down factors required for such epigenetic changes can inhibit or prevent successful reprogramming [53], [54], [56]–[59]. However, with the exception of some very early events [14], [26] the order and precise identity of chromatin modifications required for successful reprogramming is not yet well known. By precisely describing and modeling gene expression dynamics during the stochastic phase the present study provides a quantitative framework for dissecting these key rate limiting steps and will enable the mechanistic dissection of interventions known to accelerate or enhance the efficiency of the reprogramming process.

## Materials and Methods

### Production of Retrovirus

Retroviral vectors (pMIG) containing OCT4, SOX2, KLF4, c-MYC (OSKM) along with helper plasmids (VSV-G and Gag-pol) were obtained from I.H.Park (Yale University, New Haven, CT). To generate viral particles, individual retroviral vectors were co-transfected with VSV-G and Gag-pol into 293T cells seeded at 2×10^6^ cells per 10-cm^2^ using FuGENE 6 transfection reagent (Roche Applied Science). After 72-hour induction, supernatants were collected, filtered through 0.45 µm filter and concentrated using Vivaspin 300,000 MWCO PES filter columns (Sartorius). Viral titer was determined using FACS analysis for GFP expression (encoded in the pMIG vector). An MOI of 5 was used for all experiments.

### Cell Culture and Fibroblast Reprogramming

MRC-5 human fetal lung fibroblasts were obtained from I.H. Park (Yale University, New haven, CT). Briefly, MRC-5 cells were expanded in human fibroblast (hFib) media (DMEM (Gibco), 10% FBS (Milipore), 1% L-glutamine (Gibco) and 1X Penn-Strep (Gibco). One day prior to infection, 1×10^5^ MRC-5 fibroblasts were seeded into one well of a 6-well dish containing hFib media. The next day, cells were incubated in RI media (MEM alpha (Mediatech) and 10% FBS (Millipore)) containing 5 ug/mL protamine sulfate (Sigma) and OSKM virions for 24 hrs followed by replacement with fresh RI media. Cells were cultured for 72 hrs post-infection and passaged to two 10 cm^2^ dishes pre-seeded with 7.5×10^5^ inactivated feeders in hESC media supplemented with 10 µM Y-27632 (Calbiochem). After passaging, fresh hESC media was added daily until the end of the experiment. H9 human embryonic stem cells (WiCell) were maintained in hESC media (DMEM F-12 (Gibco), 20% Knockout-Serum Replacement (Gibco), 1% L-Glutamine (Gibco), 1% Non-Essential Amino Acids (Gibco), 5 µM β-mercaptoethanol (Gibco), and 2 ng/mL b-FGF) and passaged using standard methods.

### Antibody Staining and FACS Sorting of Reprogramming Cells

Reprogramming MRC-5 fibroblast cells were harvested with 1 mL Accumax (Millipore) per well (6-well dish) for 15 minutes at 37°C. Cells were pelleted, washed with PBS (Gibco) and wash buffer (2% FBS in HBSS (Invitrogen)), and resuspended in wash buffer. Cells were then stained using antibodies for SSEA4 (Biolegend, Cat# 330405) TRA-1-60 (Biolegend, Cat# 330605), washed 3 times and resuspended in FACS buffer (1% FBS in PBS). For FACS, cells were live/dead stained and gated on GFP and appropriate surface markers as indicated and single cells sorted into 96 well PCR plates. All FACS was performed using a BD Bioscience FACS Aria II.

### Quality Control and Single Cell qRT-PCR

Single cell qRT-PCR was performed as previously described [28]. Briefly, single cells were lysed and denatured by incubating at 70°C for 10 minutes and then cooled to 4°C. Cells were then reverse transcribed and pre-amplified using gene specific primers (0.25X pooled TaqMan assays) and analyzed by qPCR. qPCR was performed using TaqMan chemistry in 384 well plates on an ABI 7900 HT Fast Real-Time system. Average cycle threshold (Ct) values obtained from qPCR reactions were normalized to GAPDH (ΔCt), and inverted by taking the (40– ΔCt) value. To reduce technical error and ensure robust sample quality, all cells with a GAPDH Ct value of 25 or greater were excluded from further analysis. TaqMan assays for endogenous OCT4, SOX2, KLF4 and c-MYC were directed against the 3′-UTR region of the transcript, which is distinct from the synthetic UTRs incorporated in the viral OSKM transgenes, conferring their specificity to the endogenous transcripts.

### Marker Panel Selection

Genes selected for inclusion in our 48 marker panel were chosen based on several criteria. For pluripotency and chromatin modifier genes we selected those whose role in the establishment or maintenance of the pluripotent state was well documented and experimentally validated. This decision was further informed using the dataset of Dowell et. al. [60] which assigns a self-renewal score to genes based on their integration in the pluripotency gene regulatory network (as determined by direct binding of O, S, K and/or M) as well as their degree of co-expression with well-established pluripotency genes. Fibroblast genes were selected based on their expression in fibroblasts and absence from hESCs as determined in [32], [61].

### Data Analysis

Distance was determined by reducing gene expression to 0(undetected) and 1(detected, Ct <40) and calculating the average Euclidean distance for each cell to the FIB and PLURI groups, ignoring self-comparisons. Similarity was computed for each group distance by taking the ratio of the distance between FIB and PLURI minus each cell’s distance to the group in question, over the distance between FIB and PLURI minus the average distance of that group to itself. The average of the similarity to PLURI and the complement of the similarity to FIB was taken as an estimate of the progression of each cell along the PLURI trajectory. Distance off of the trajectory was taken as the Euclidean distance from the FIB and PLURI similarities to the trajectory value.

PCA-based SOM analysis was performed in JMP, Version 10 (a SAS product) [62] using a 5-by-1 matrix and visualizing on a biplot (PC1 vs PC2). Cells within the “Alt” group were considered to be outliers (as described above) and were excluded from subsequent analysis, unless otherwise indicated. Hierarchical clustering was also performed in JMP, using Ward’s method with no standardization, on (40-ΔCT) values. Coverage ellipses on the Euclidian distance graphs represent 90% coverage of the data points from the group indicated. For correlation analysis Pearson’s correlation coefficients within a defined SOM grouping were taken for the entire 48×48 matrix of genes analyzed in this study. Network graphs were constructed in Cytoscape using a force-directed layout derived from the top 100 Pearson correlations between all of the cells, excluding outliers, in our analysis (n = 117).

### Model Generation

To generate accurate models, the data was first interpolated to generate a high resolution training set. The entire sample population was included, except for outliers considered as the cells with the highest distance off of the trajectory (10%, N = 17). The training data represented the percentage of cells expressing a gene at any point along the PLURI trajectory, and was measured by uniformly placing overlapping bins of fixed width across the range and directly counting the number of cells expressing each gene. Models were generated to then predict the percentage expressing at any trajectory location. ‘Uniform’ models were generated by assigning a ‘Baseline’ value at the start of the trajectory ( = 0), and fixing a slope such that a straight line passed from the ‘Baseline’ to the value at the end of the trajectory ( = 1). ‘Normal’ models were then fit to this data using the ‘optim’ function in R, attempting to minimize the mean squared error, using the constraint, 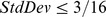 and the following form:
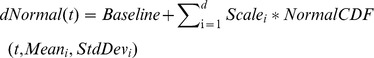



In order to verify model quality and compare fitting between different models, AICc was calculated and a bootstrapping test was performed. AICc was calculated by:
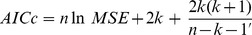
where *n* is the effective number of sample points present in the original data, *k* is the number of free model parameters, and *MSE* is the mean squared error from the model prediction to the training data. Bootstrapping was performed by repeatedly simulating the training data but using only *n* bins and randomly resampling a fixed number of cells from each bin’s range. The error between the model prediction and the resampled data was compared to the expected error using an F-test to predict if the error induced by lack-of-fit exceeded the pure error of the data by a significant level, and this was tracked as a percentage of all tests done against the model.

### Correlation Analysis

First, simulated populations of an equal size were generated by sampling a set of points along the reprogramming progression axis such that they matched the distribution of values in the original dataset. For each sampling point, representative of a single simulated cell, each gene was set to detected or undetected independently, using the frequency curves generated from our Gaussian model. Pearson correlation coefficients were then computed for this reference population, and averaged over repeated runs (n = 1000000). Differences in correlation between this background dataset and those calculated for our observed data were then tested for significance using the ‘r.test’ function of the R package ‘psych’.

## Supporting Information

Figure S1 (PDF)

Figure S2 (PDF)

Figure S3 (PDF)

Figure S4 (PDF)

Figure S5 (PDF)

Figure S6 (PDF)

Table S1 (PDF)

Table S2 (XLSX)

Table S3 (PDF)

Table S4 (XLSX)
