# Supplementary material for: Single Cell Analysis Reveals the Stochastic Phase of Reprogramming to Pluripotency Is an Ordered Probabilistic Process
Source: PLoS One. 2014 Apr 17;9(4):e95304. doi: 10.1371/journal.pone.0095304 (PMC3990627; doi:10.1371/journal.pone.0095304)
Supplement: Figure S1 — (PDF) [file pone.0095304.s001.pdf]

Figure S1

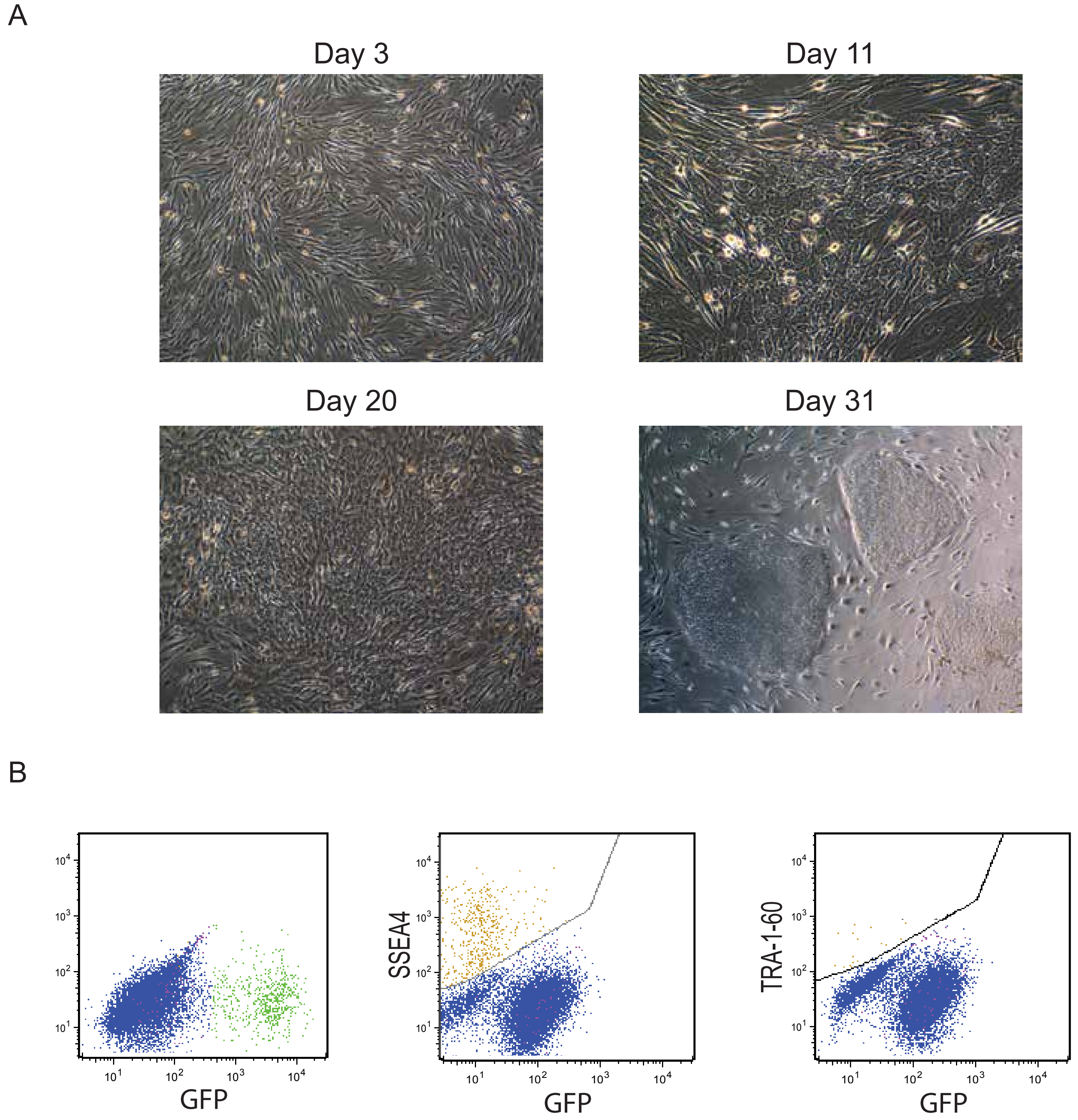

Figure S1: Overview of Experimental Design
(A) Representative images of Human MRC-5 cells undergoing reprogramming at indicated time points post-infection with OSKM virus. (B) Representative FACS plots showing the gating scheme used for the isolation of GFP⁺, SSEA4⁺ and TRA-1-60⁺ cells.
