# Supplementary material for: Single Cell Analysis Reveals the Stochastic Phase of Reprogramming to Pluripotency Is an Ordered Probabilistic Process
Source: PLoS One. 2014 Apr 17;9(4):e95304. doi: 10.1371/journal.pone.0095304 (PMC3990627; doi:10.1371/journal.pone.0095304)
Supplement: Figure S2 — (PDF) [file pone.0095304.s002.pdf]

Figure S2

Pluripotency

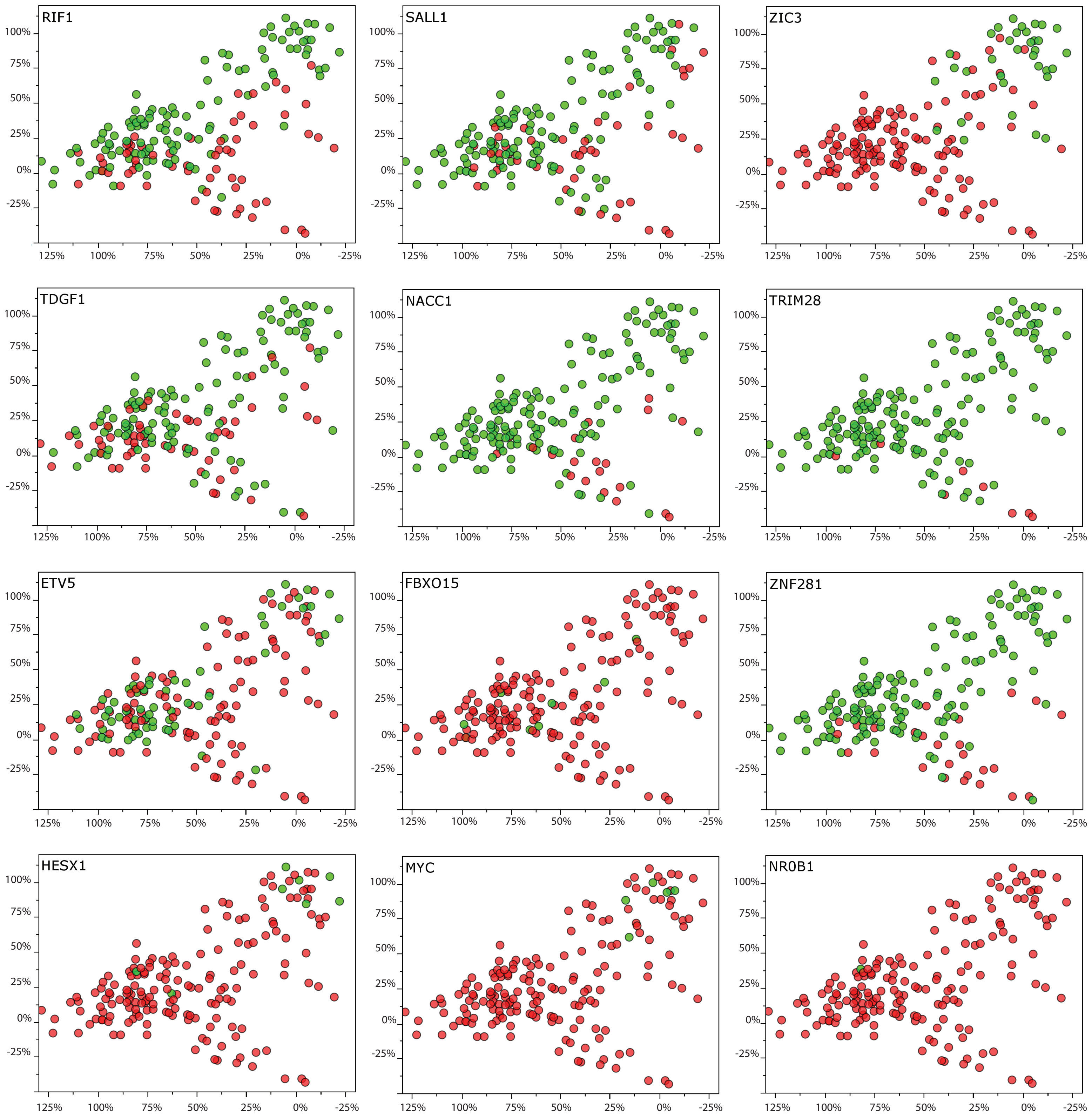

Pluripotency

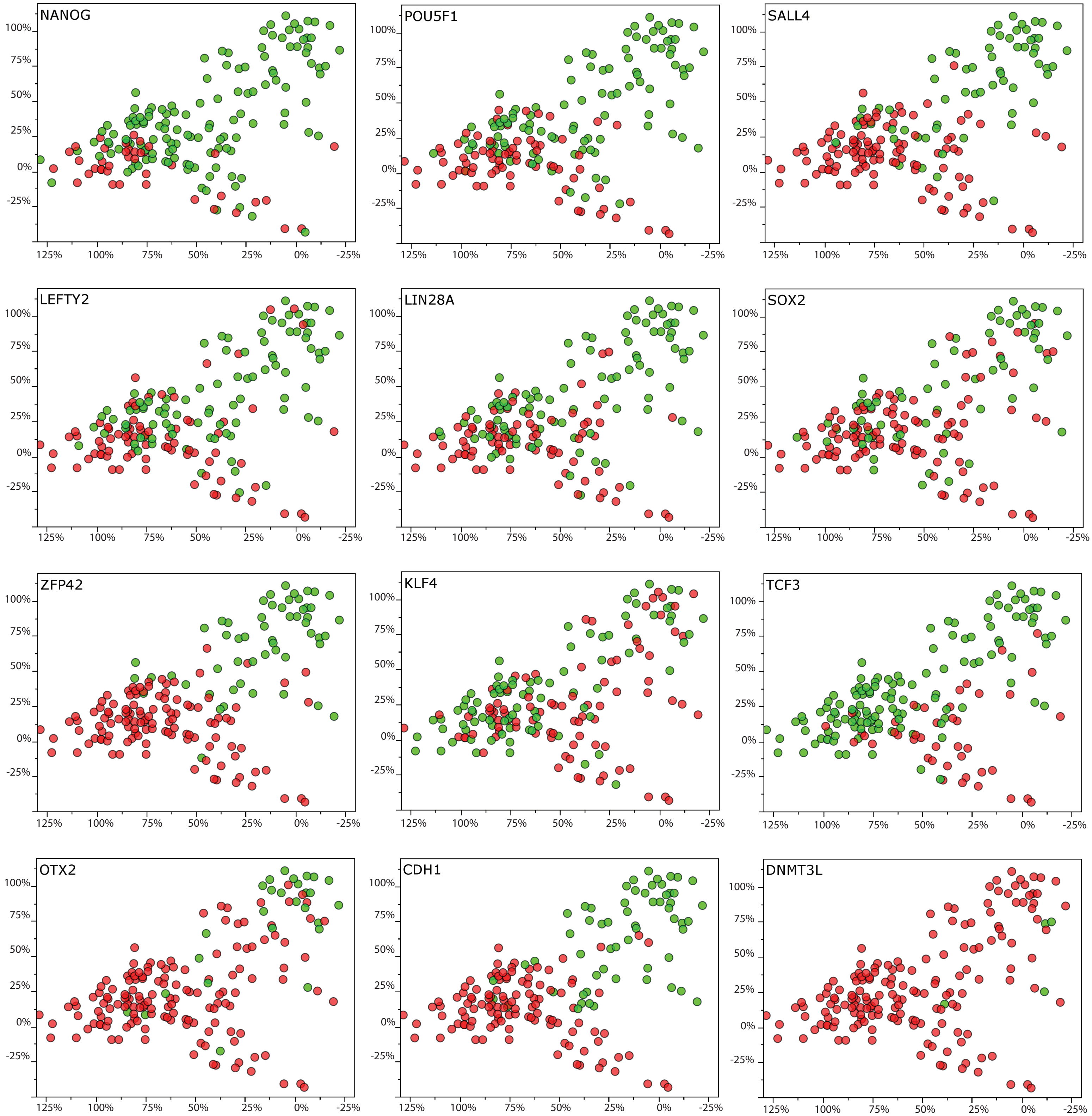

Fibroblast

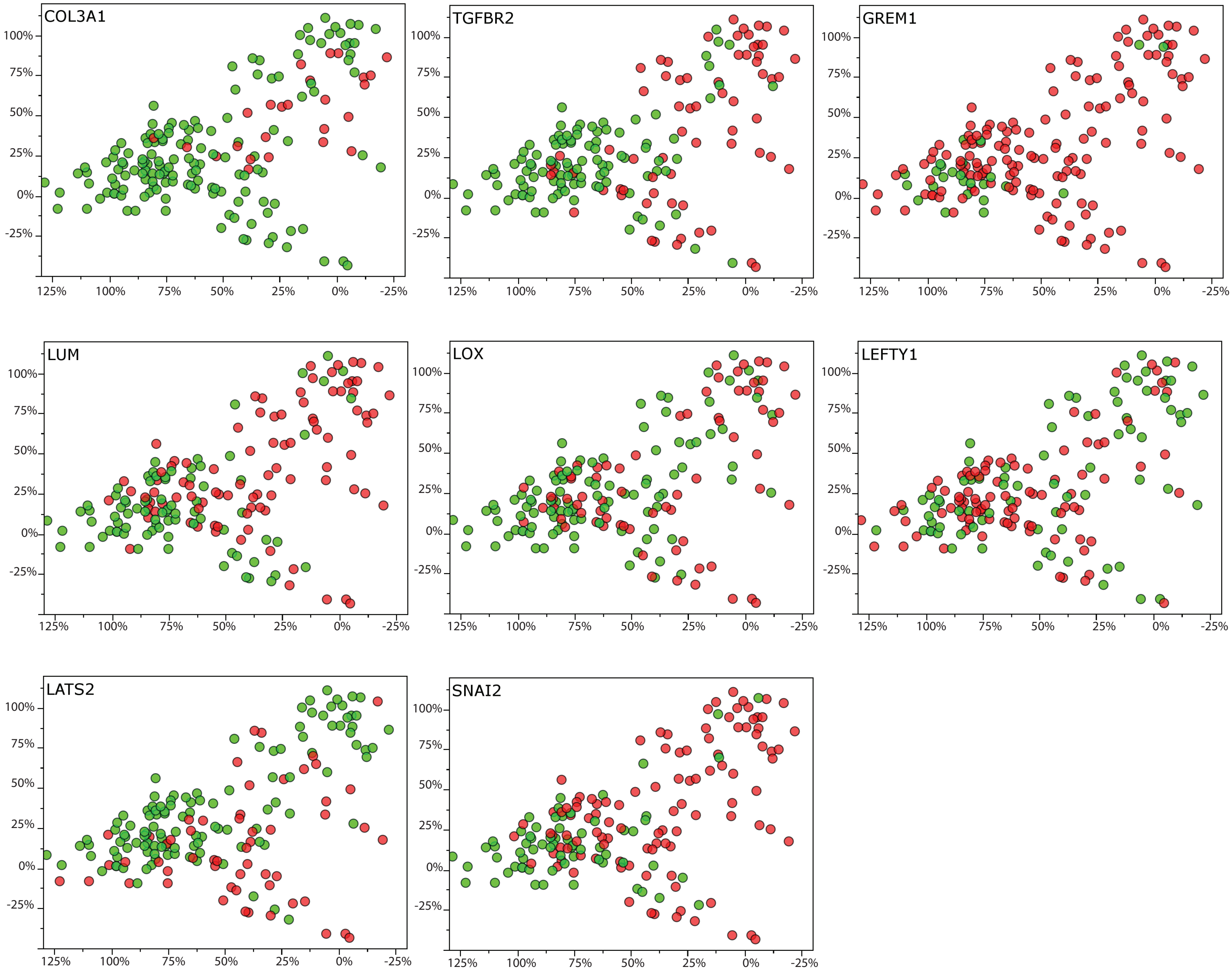

Chromatin Modifiers

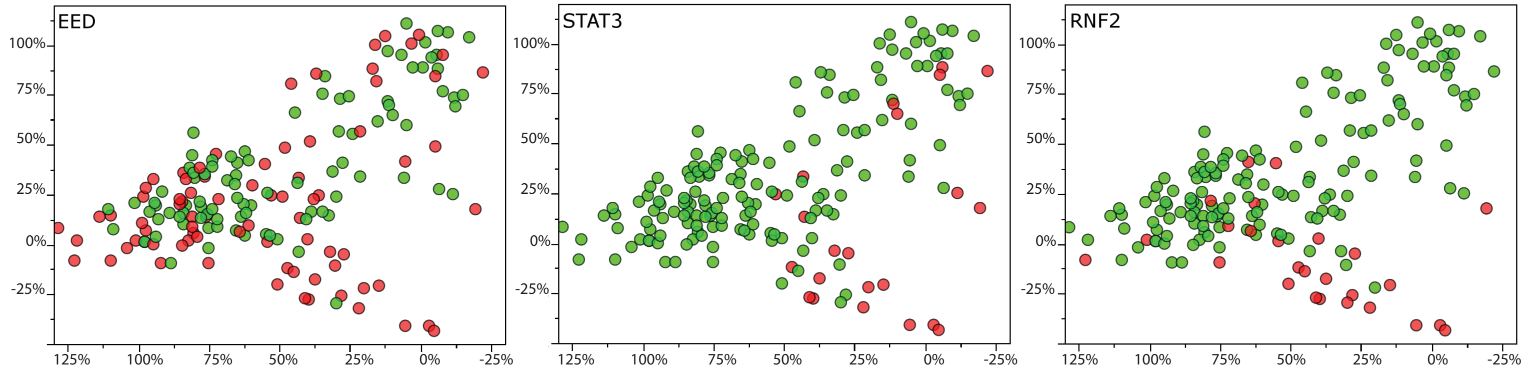

Chromatin Modifiers

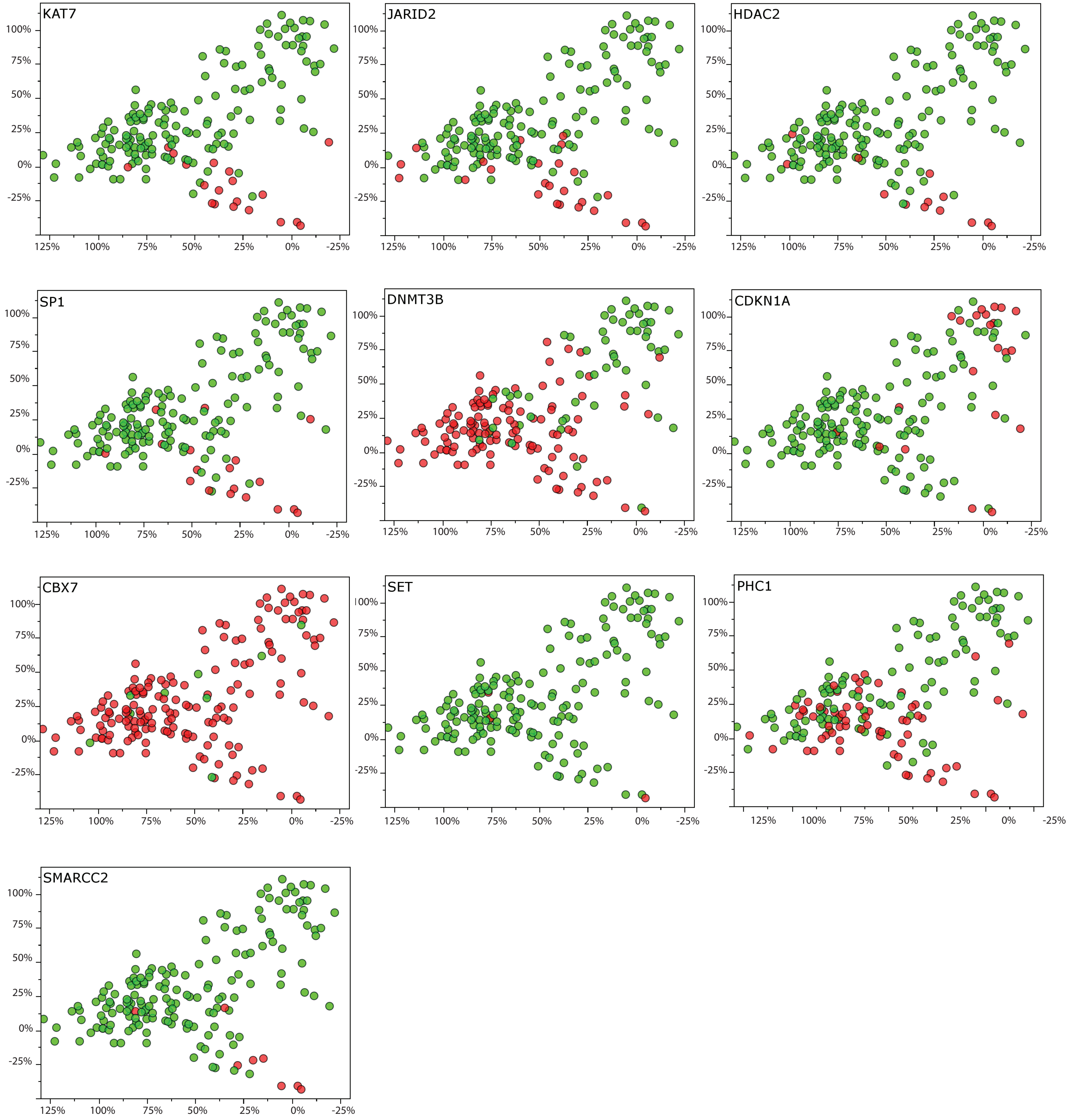

Intermediate

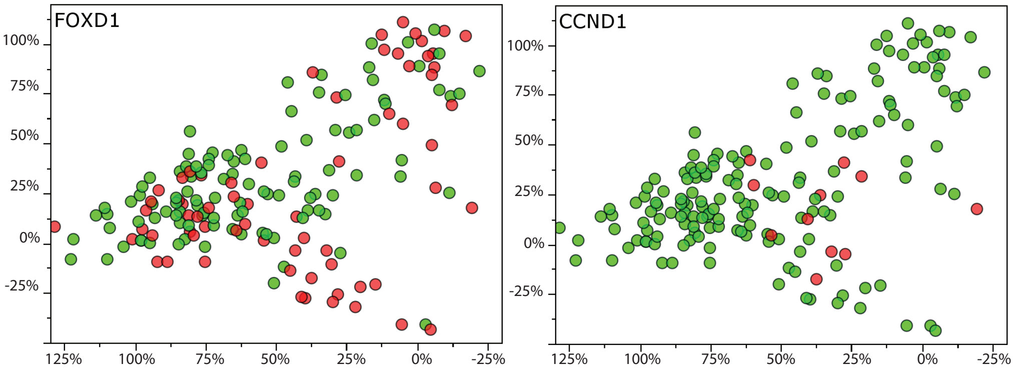

Figure S3: Bubble Plots Demonstrating Qualitative Changes in Gene Expression During Reprogramming
Bubble plots were generated using the relative fibroblast and relative H9 similarity metrics to plot the presence (green) or absence (red) of genes expression in a given cell. This view of the transcriptional dynamics during reprogramming reveals genes activated early, intermediately, or late in the process. Genes with no qualitative changes in expression are also observed.
