# Supplementary material for: Single Cell Analysis Reveals the Stochastic Phase of Reprogramming to Pluripotency Is an Ordered Probabilistic Process
Source: PLoS One. 2014 Apr 17;9(4):e95304. doi: 10.1371/journal.pone.0095304 (PMC3990627; doi:10.1371/journal.pone.0095304)
Supplement: Figure S3 — (PDF) [file pone.0095304.s003.pdf]

Figure S3

Fibroblast

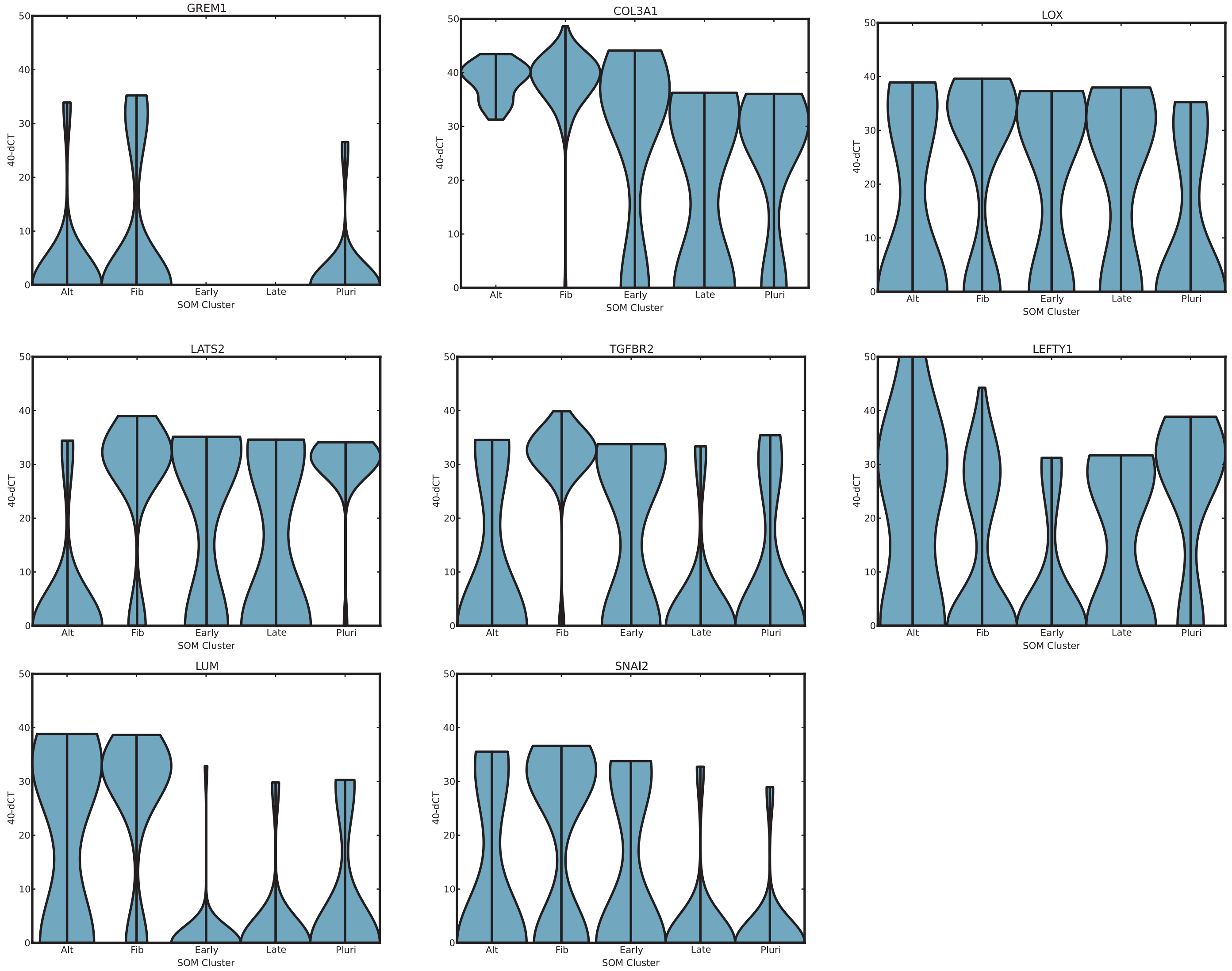

Intermediate

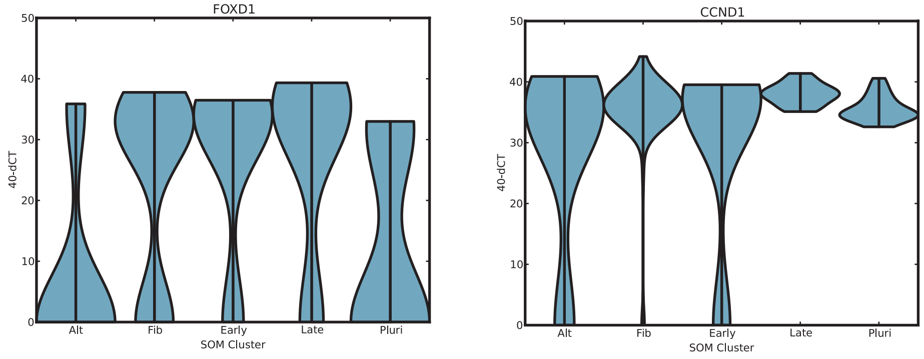

Chromatin Modifiers / Cell Cycle

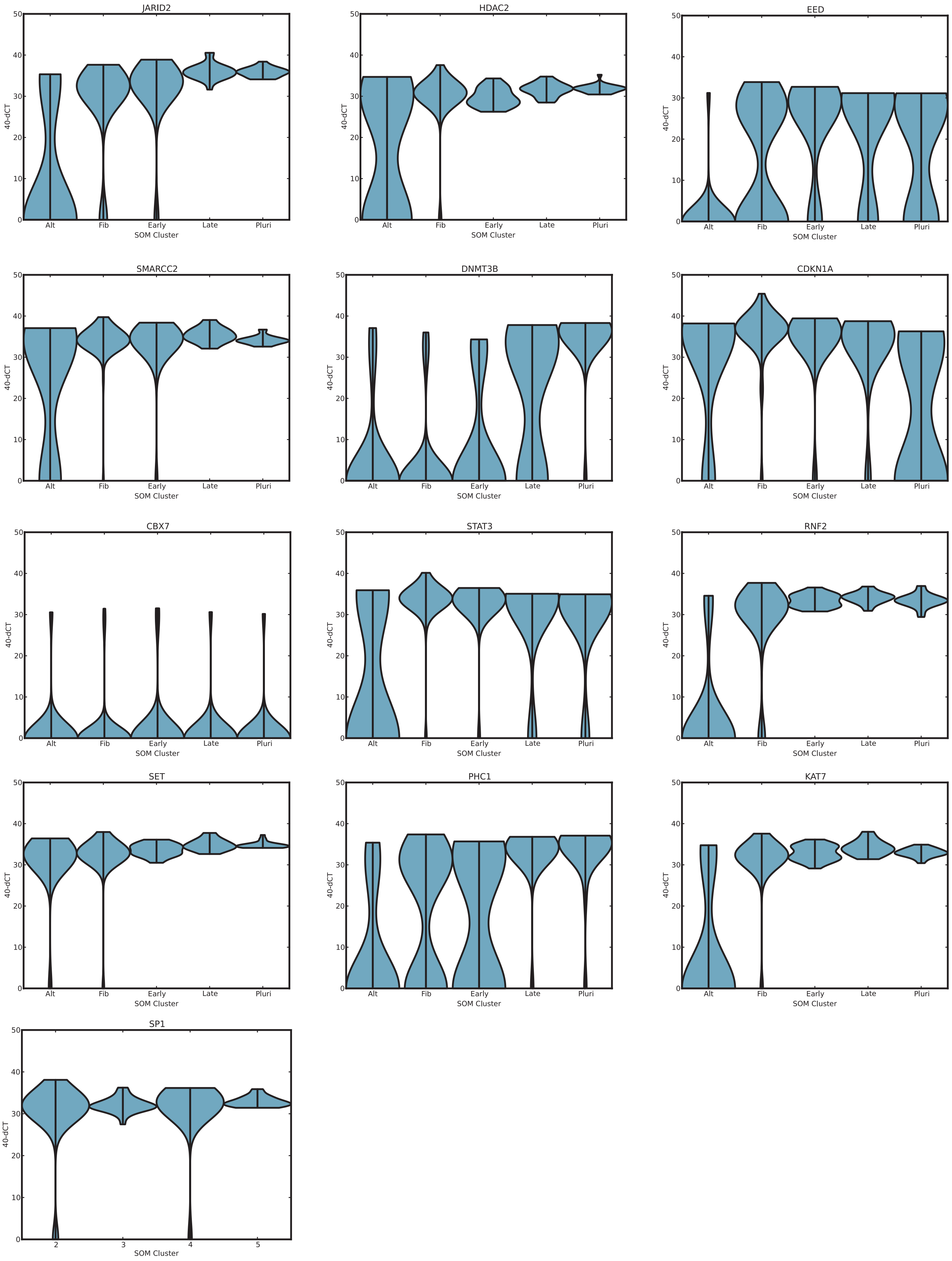

Pluripotency

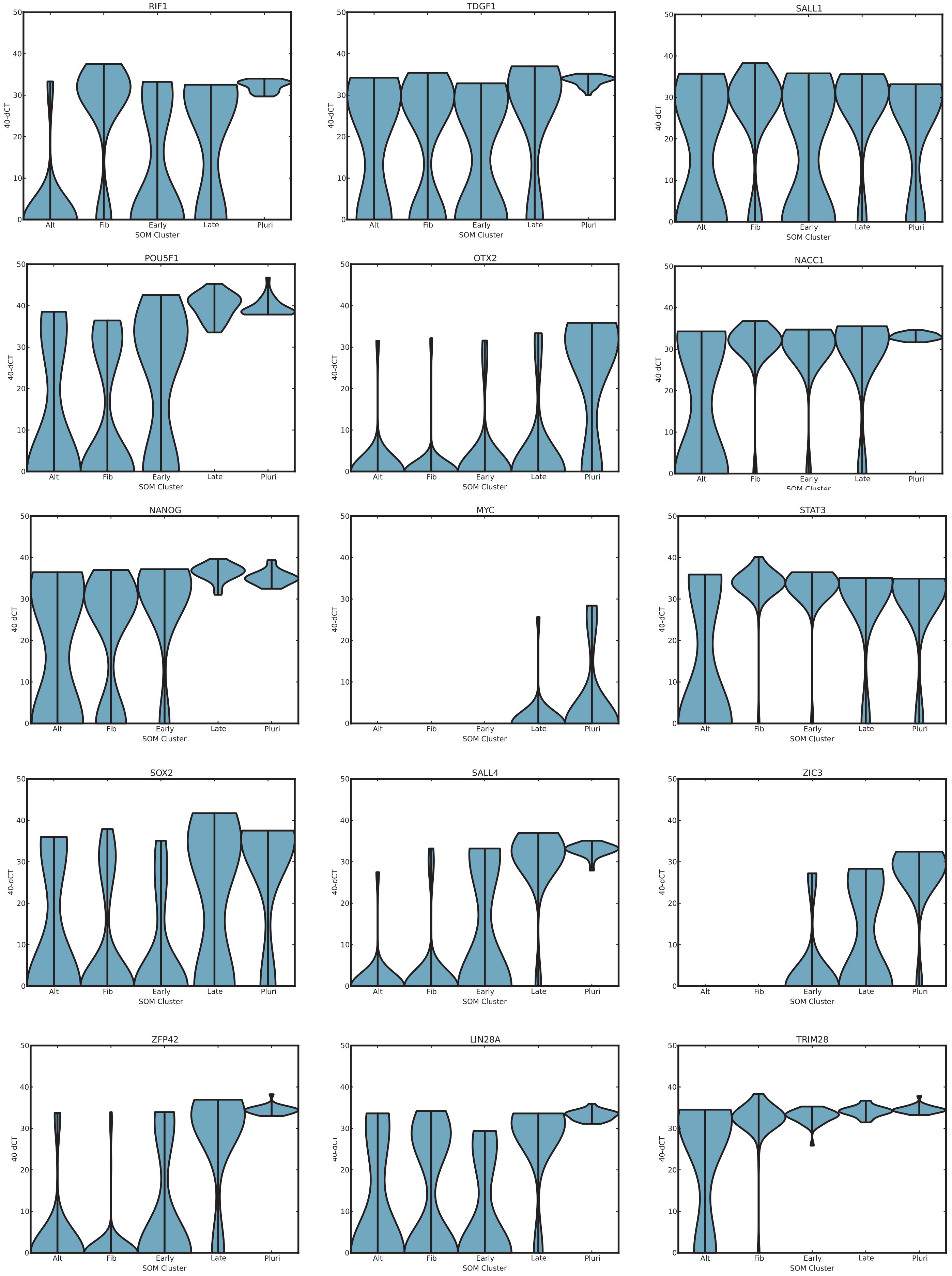

Pluripotentcy (cont.)

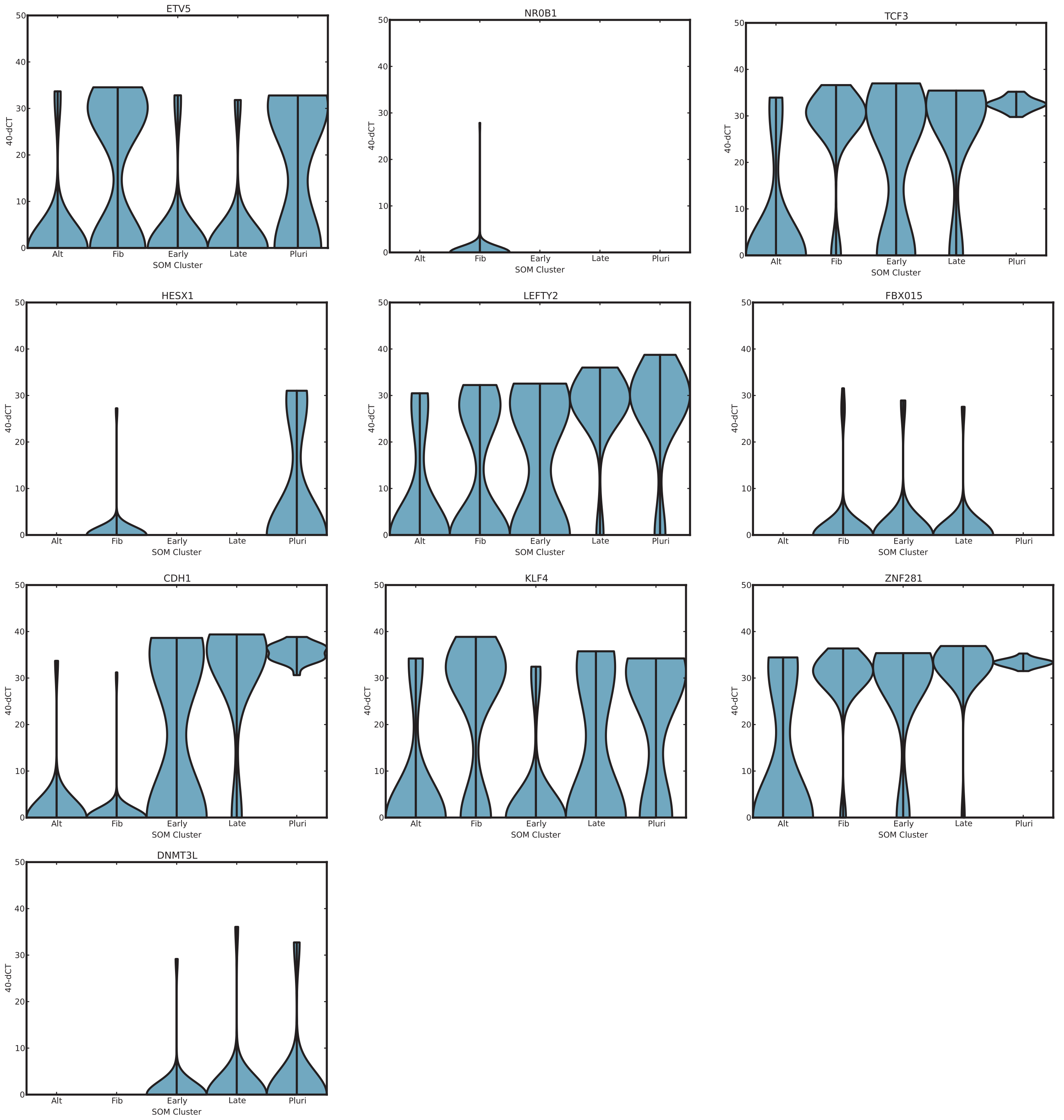

Figure S3: Violin Plots Depicting Quantitative Changes in Gene Expression During Reprogramming

Cells were separated by SOM grouping (outliers excluded) and gene expression levels were plotted for each cell within the group. The width of the violin represents the distribution of cells across expression levels. For the majority of genes, there is a clear inflection point in these graphs where a large number of cellsup or down regulate a given transcript when transitioning from one SOM group to another, eventually coalescing around high or low expression levels as cells approach pluripotency and cellular phenotypes stabilize.
