# Supplementary material for: Single Cell Analysis Reveals the Stochastic Phase of Reprogramming to Pluripotency Is an Ordered Probabilistic Process
Source: PLoS One. 2014 Apr 17;9(4):e95304. doi: 10.1371/journal.pone.0095304 (PMC3990627; doi:10.1371/journal.pone.0095304)
Supplement: Figure S4 — (PDF) [file pone.0095304.s004.pdf]

Figure S4

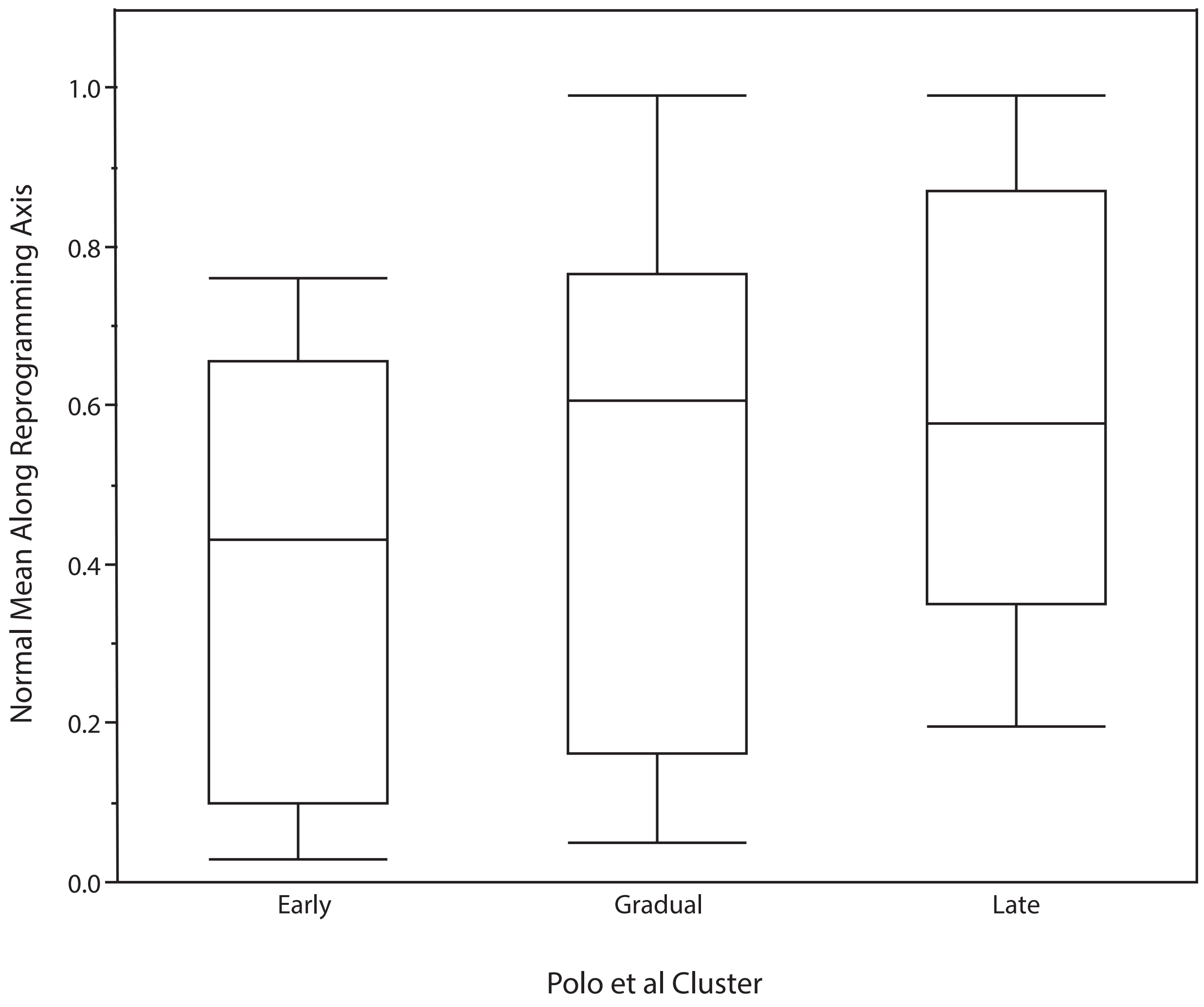

Figure S4: Comparison of timing of gene activation / inactivation with Polo et all 2012. Using the cluster definitions provided in Polo et al genes whose expression increased or decreased Early, Gradually or Late were collapsed into a single Early, Gradual or Late cluster. We then compared these clusters to the mean of the normal distribution for each gene as defined in our model as shown in the box and whisker plots above.
