# Supplementary material for: Single Cell Analysis Reveals the Stochastic Phase of Reprogramming to Pluripotency Is an Ordered Probabilistic Process
Source: PLoS One. 2014 Apr 17;9(4):e95304. doi: 10.1371/journal.pone.0095304 (PMC3990627; doi:10.1371/journal.pone.0095304)
Supplement: Figure S5 — (PDF) [file pone.0095304.s005.pdf]

Figure S5

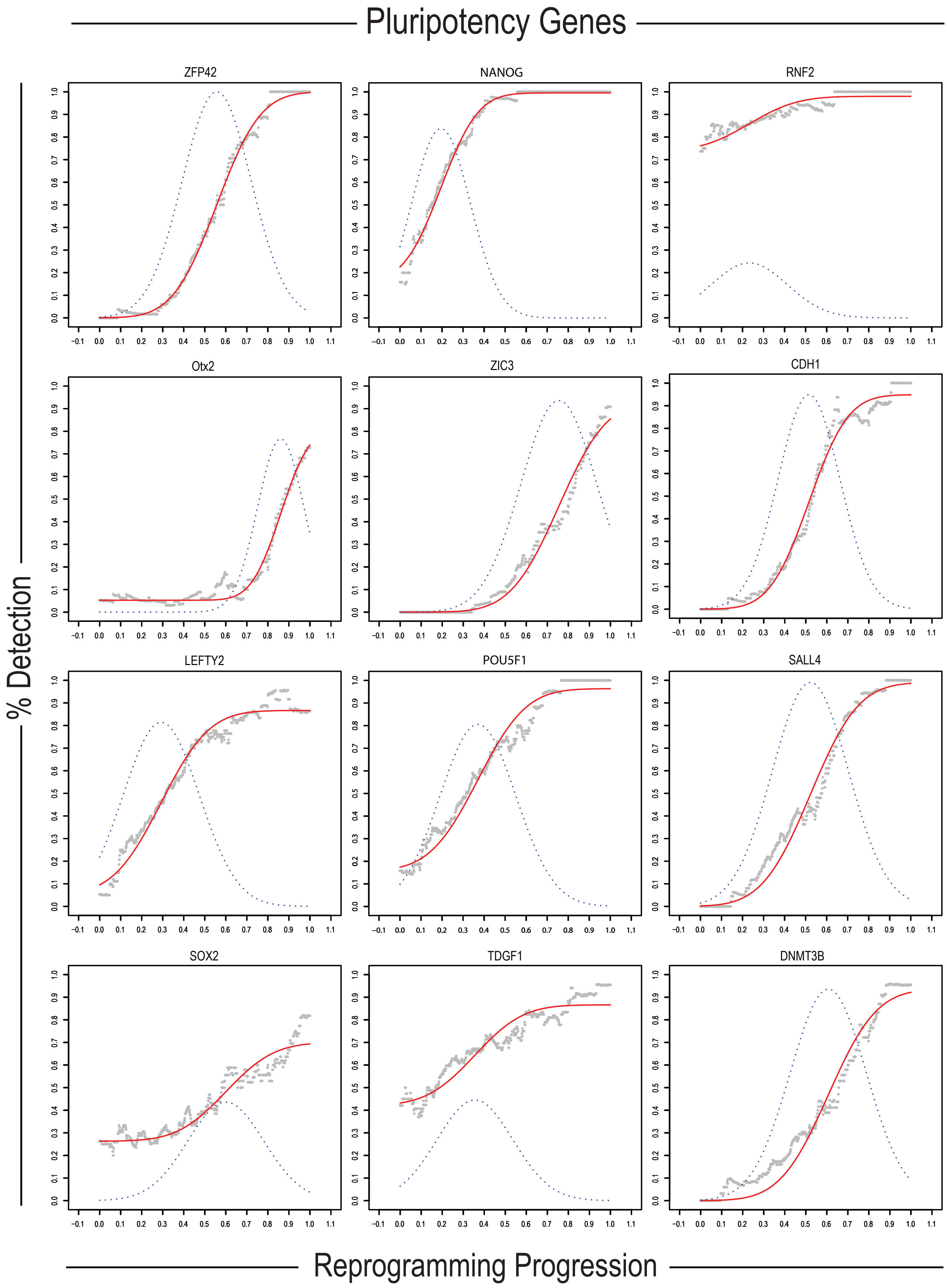

Pluripotency Genes
ZFP42
NANOG
RNF2
Otx2
ZIC3
CDH1
LEFTY2
POU5F1
SALL4
SOX2
TDGF1
DNMT3B
% Detection
Reprogramming Progression

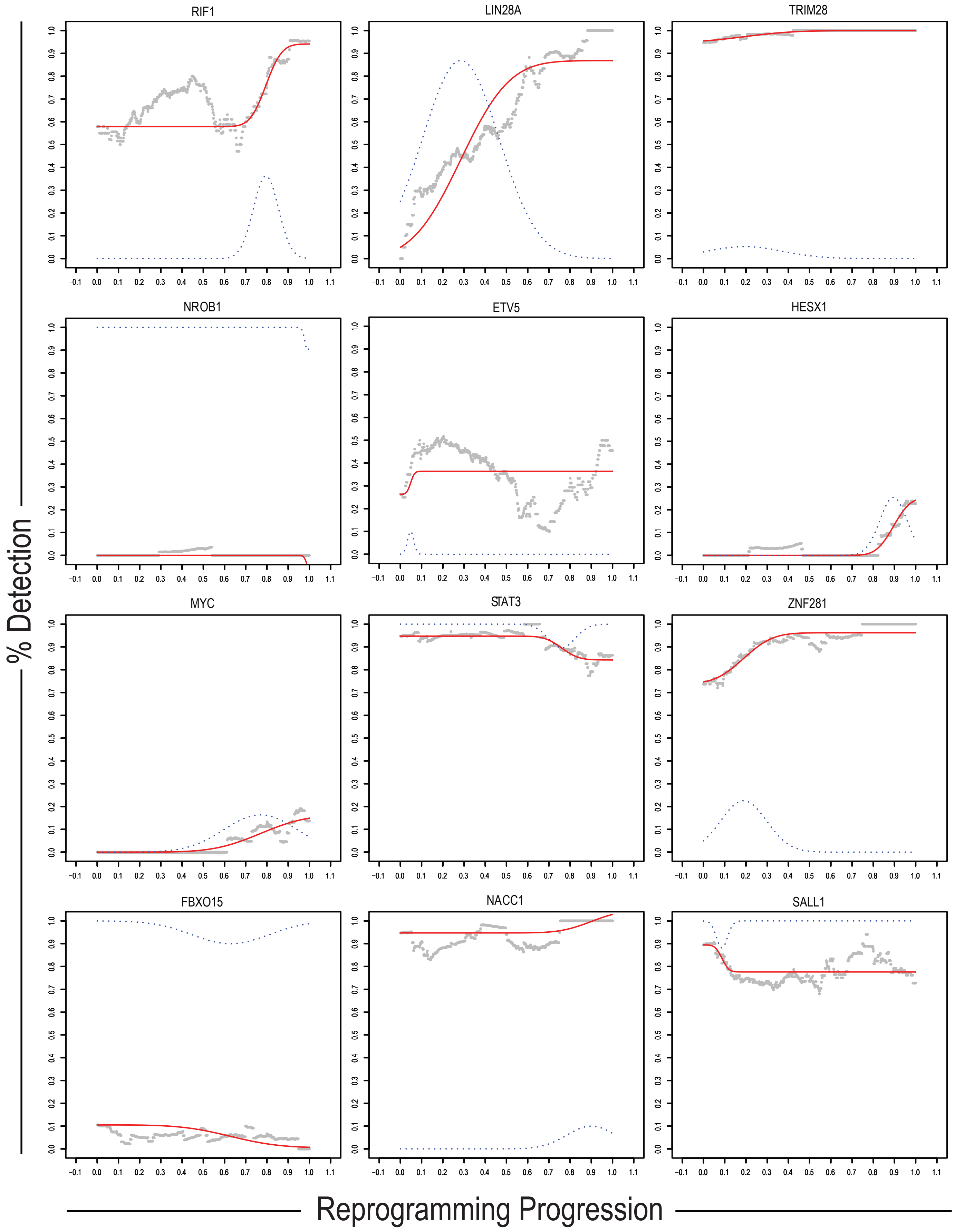
RIF1
LIN28A
TRIM28
NROB1
ETV5
HESX1
MYC
STAT3
ZNF281
FBXO15
NACC1
SALL1
% Detection
Reprogramming Progression

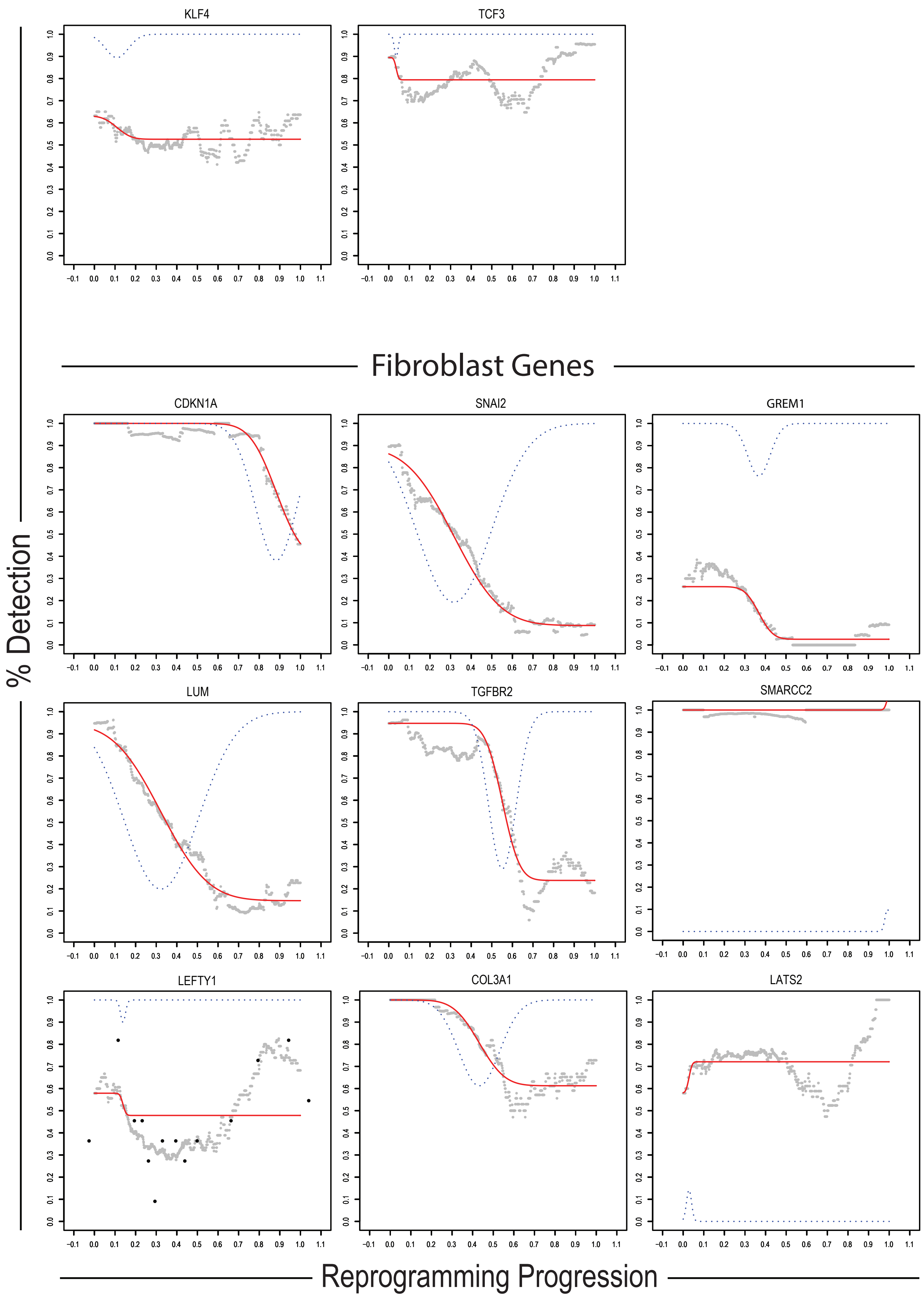
KLF4
TCF3
Fibroblast Genes
CDKN1A
SNAI2
GREM1
LUM
TGFBR2
SMARCC2
LEFTY1
COL3A1
LATS2
% Detection
Reprogramming Progression

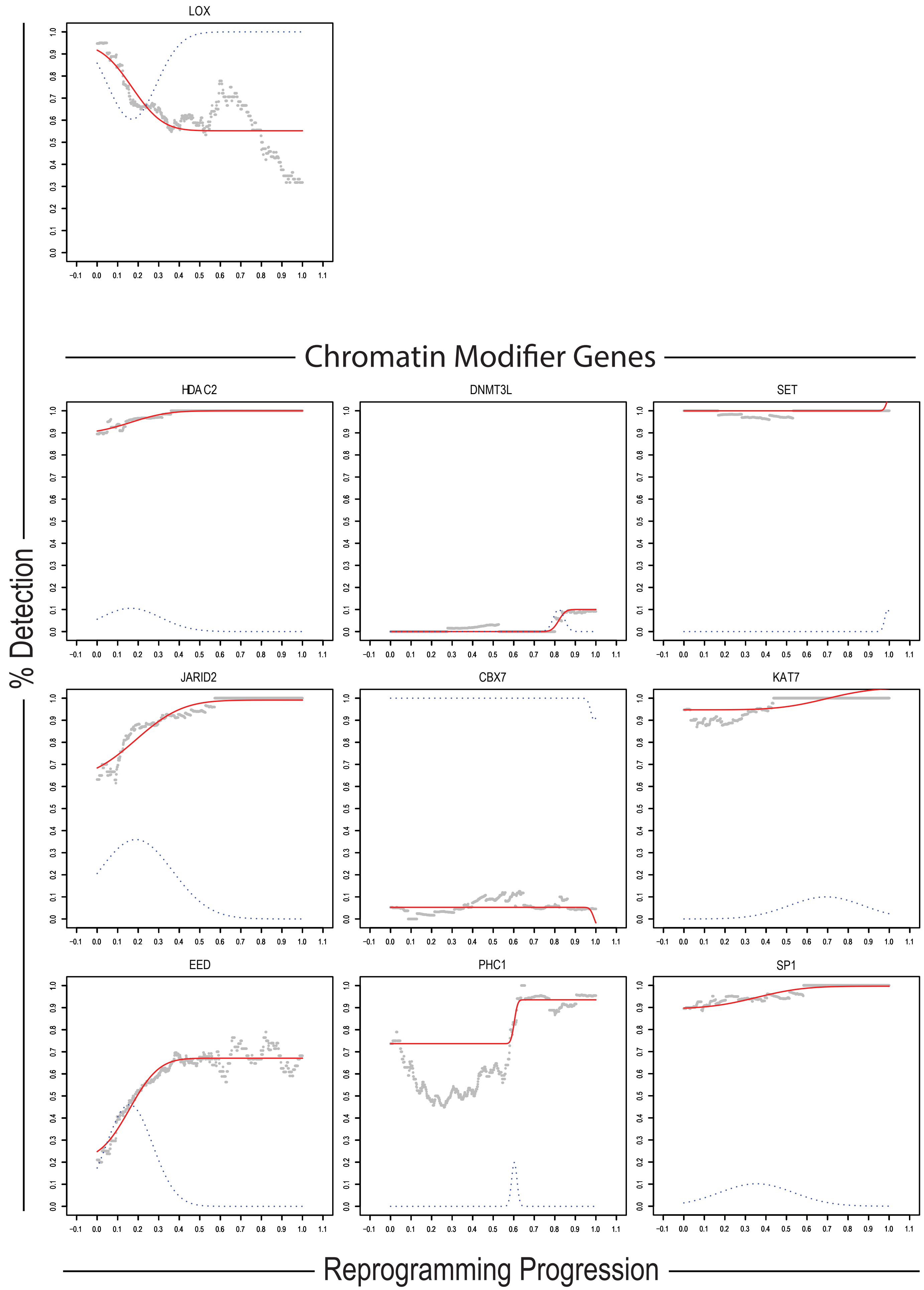
LOX
Chromatin Modifier Genes
HDAC2
DNMT3L
SET
JARID2
CBX7
KAT7
EED
PHC1
SP1
% Detection
Reprogramming Progression

Figure S5: Modeling Gene Expression Dynamics Using Gaussian Distributions
Models depict the observed detection frequency (grey dots) along the Reprogramming Progression Axis using a sliding window analysis as described in Methods. Red lines depict the model fit resulting from the underlying normal distribution (blue dotted line).
