# Supplementary material for: Single Cell Analysis Reveals the Stochastic Phase of Reprogramming to Pluripotency Is an Ordered Probabilistic Process
Source: PLoS One. 2014 Apr 17;9(4):e95304. doi: 10.1371/journal.pone.0095304 (PMC3990627; doi:10.1371/journal.pone.0095304)
Supplement: Figure S6 — (PDF) [file pone.0095304.s006.pdf]

Figure S6

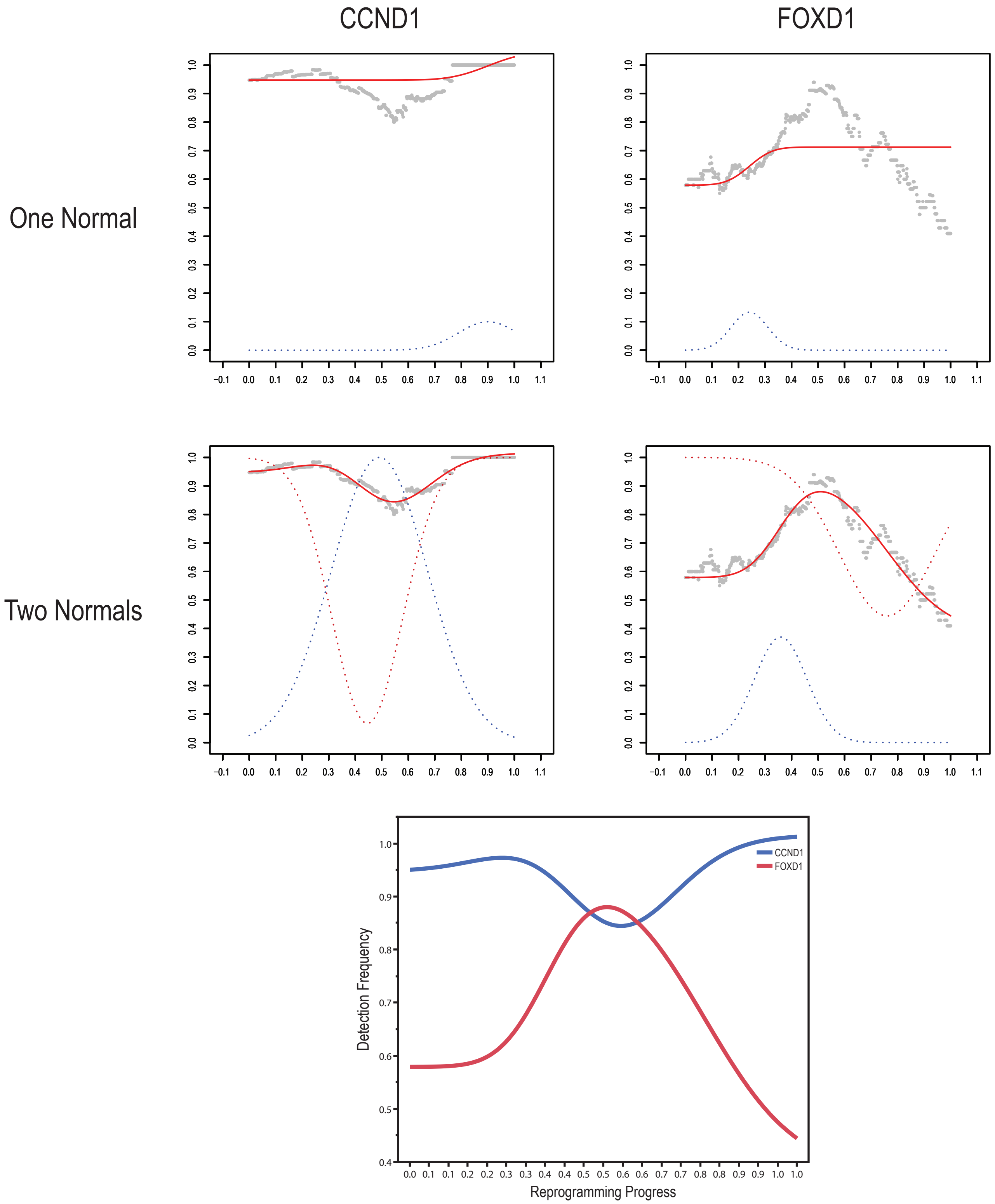

Figure S6: Modeling Complex Gene Behavior with Two Gaussian Distributions
Genes with complex behavior (transiently activated or inactivated) were modeled using one (top panel) or two (bottom panel) Gaussian distributions. Poor fit is observed using a single Gaussian curve, however accounting for both activating (blue dotted line) and repressive (red dotted line) events using two curves results in excellent fit to the data. A combined view of these expression probabilities is shown in the bottom panel.
