# Supplementary material for: Single Cell Analysis Reveals the Stochastic Phase of Reprogramming to Pluripotency Is an Ordered Probabilistic Process
Source: PLoS One. 2014 Apr 17;9(4):e95304. doi: 10.1371/journal.pone.0095304 (PMC3990627; doi:10.1371/journal.pone.0095304)
Supplement: Table S1 — (PDF) [file pone.0095304.s007.pdf]

Table S1

Gene Name	Taqman Assay ID
CBX7	Hs00545603_m1
CCND1	Hs00765553_m1
CDH1	Hs01023894_m1
CDKN1A	Hs00355782_m1
COL3A1	Hs00943809_m1
DNMT3B	Hs00171876_m1
DNMT3L	Hs01081364_m1
EED	Hs00537777_m1
ETV5	Hs00231790_m1
FBXO15	Hs00380856_m1
FOXD1	Hs00270117_s1
GAPDH	Hs99999905_m1
GREM1	Hs01879841_s1
HDAC2	Hs00231032_m1
HESX1	Hs00172696_m1
JARID2	Hs01004460_m1
KAT7 (MYST2)	Hs01561260_m1
KLF4	Custom
LATS2	Hs00324396_m1
LEFTY1	Hs00764128_s1
LEFTY2	Hs00745761_s1
LIN28A	Hs00702808_s1
LOX	Hs00942480_m1
LUM	Hs00158940_m1
MYC	Hs01570247_m1
NACC1	Hs00369413_m1
NANOG	Hs02387400_g1
NR0B1(DAX1)	Hs03043658_m1
OTX2	Hs00222238_m1
PHC1	Hs01051497_m1
POU5F1	Custom
REST	Hs00958503_m1
RIF1	Hs00871714_m1
RNF2	Hs00200541_m1
SALL1	Hs00231307_m1
SALL4	Hs00360675_m1
SET	Hs00853870_g1
SMARCC2	Hs00161961_m1
SNAI2	Hs00950344_m1
SOX2	Hs01053049_s1
SP1	Hs00916521_m1
STAT3	Hs01047580_m1
TCF3	Hs01012685_m1
TDGF1	Hs02339499_g1
TGFBR2	Hs00234253_m1
TRIM28	Hs00232212_m1
ZFP42 (REX1)	Hs00399279_m1
ZIC3	Hs00185665_m1
ZNF281	Hs00273550_s1

Table S1: List of 48 Taqman Assays Used for Single-Cell qRT-PCR
