# Supplementary material for: Single Cell Analysis Reveals the Stochastic Phase of Reprogramming to Pluripotency Is an Ordered Probabilistic Process
Source: PLoS One. 2014 Apr 17;9(4):e95304. doi: 10.1371/journal.pone.0095304 (PMC3990627; doi:10.1371/journal.pone.0095304)
Supplement: Table S3 — (PDF) [file pone.0095304.s009.pdf]

Table S3

	FIBROBLAST	GFP+ DAY 4	GFP+ DAY 8	GFP+ DAY 14	SSEA4+ DAY 4	SSEA4+ DAY 8	SSEA4+ DAY 14	TRA-1-60+	CDH1+	HESC
**# USED IN ANALYSIS**	15	14	15	15	16	16	13	48	16	15

Table S3: Phenotype and number of cells collected by FACS and used for analysis
